# The phenotypic and genetic association between endometriosis and immunological diseases

**DOI:** 10.1093/humrep/deaf062

**Published:** 2025-04-22

**Authors:** Nina Shigesi, Holly R Harris, Hai Fang, Anne Ndungu, Matthew R Lincoln, The International Endometriosis Genome Consortium, The 23andMe Research Team, Chris Cotsapas, Julian Knight, Stacey A Missmer, Andrew P Morris, Christian M Becker, Nilufer Rahmioglu, Krina T Zondervan

**Affiliations:** Oxford Endometriosis CaRe Centre, Nuffield Department of Women’s and Reproductive Health, John Radcliffe Hospital, University of Oxford, Oxford, UK; Public Health Sciences Division, Fred Hutchinson Cancer Center, Seattle, WA, USA; Department of Epidemiology, School of Public Health, University of Washington, Seattle, WA, USA; Centre for Human Genetics, University of Oxford, Oxford, UK; Shanghai Institute of Hematology, State Key Laboratory of Medical Genomics, National Research Center for Translational Medicine at Shanghai, Ruijin Hospital, Shanghai Jiao Tong University School of Medicine, Shanghai, China; Centre for Human Genetics, University of Oxford, Oxford, UK; Institute of Medical Sciences, University of Toronto, Toronto, ON, Canada; The International Endometriosis Genome Consortium; 23andMe, Inc., Sunnyvale, CA, USA; Center for Neurocognition and Behavior/Center for Neurodevelopment and Plasticity, Wu Tsai Institute, Yale University, New Haven, CT, USA; Centre for Human Genetics, University of Oxford, Oxford, UK; Department of Epidemiology, Harvard T.H. Chan School of Public Health, Boston, MA, USA; Department of Obstetrics and Gynecology, University of Michigan, Ann Arbor, MI, USA; Centre for Genetics and Genomics Versus Arthritis, Centre for Musculoskeletal Research, The University of Manchester, Manchester, UK; Oxford Endometriosis CaRe Centre, Nuffield Department of Women’s and Reproductive Health, John Radcliffe Hospital, University of Oxford, Oxford, UK; Oxford Endometriosis CaRe Centre, Nuffield Department of Women’s and Reproductive Health, John Radcliffe Hospital, University of Oxford, Oxford, UK; Centre for Human Genetics, University of Oxford, Oxford, UK; Oxford Endometriosis CaRe Centre, Nuffield Department of Women’s and Reproductive Health, John Radcliffe Hospital, University of Oxford, Oxford, UK; Centre for Human Genetics, University of Oxford, Oxford, UK

**Keywords:** endometriosis, immune conditions, osteoarthritis, rheumatoid arthritis, multiple sclerosis, coeliac disease, psoriasis, genetics, GWAS

## Abstract

**STUDY QUESTION:**

Is there an increased risk of immunological diseases among endometriosis patients, and does a shared genetic basis contribute to this risk?

**SUMMARY ANSWER:**

Endometriosis patients show a significantly increased risk of autoimmune, autoinflammatory, and mixed-pattern diseases, including rheumatoid arthritis, multiple sclerosis, coeliac disease, osteoarthritis, and psoriasis, with genetic correlations between endometriosis and osteoarthritis, rheumatoid arthritis, and multiple sclerosis, and a potential causal link to rheumatoid arthritis.

**WHAT IS KNOWN ALREADY:**

The epidemiological evidence for an increased risk of immunological diseases among women with endometriosis is limited in scope and has varied in robustness due to the opportunity for biases. The presence of a biological basis for increased comorbidity across immunological conditions has not been investigated. Here we investigate the phenotypic and genetic association between endometriosis and 31 immune conditions in the UK Biobank.

**STUDY DESIGN, SIZE, DURATION:**

Phenotypic analyses between endometriosis and immune conditions (17 classical autoimmune, 10 autoinflammatory, and 4 mixed-pattern diseases) were conducted using two approaches (8223 endometriosis, 64 620 immunological disease cases): (i) retrospective cohort study design to incorporate temporality between diagnoses and (ii) cross-sectional analysis for simple association. Genome-wide association studies (GWAS) and meta-analyses for those immune conditions that showed phenotypic association with endometriosis (1493–77 052 cases) were conducted.

**PARTICIPANTS/MATERIALS, SETTING, METHODS:**

Comprehensive phenotypic association analyses were conducted in females in the UK Biobank. GWAS for immunological conditions were conducted in females-only and sex-combined study populations in UK Biobank and meta-analysed with existing largest available GWAS results. Genetic correlation and Mendelian randomization (MR) analyses were conducted to investigate potential causal relationships. Those immune conditions with significant genetic correlation with endometriosis were included in multi-trait analysis of GWAS to boost discovery of novel and shared genetic variants. These shared variants were functionally annotated to identify affected genes utilizing expression quantitative trait loci (eQTL) data from GTEx and eQTLGen databases. Biological pathway enrichment analysis was conducted to identify shared underlying biological pathways.

**MAIN RESULTS AND THE ROLE OF CHANCE:**

In both retrospective cohort and cross-sectional analyses, endometriosis patients were at significantly increased (30–80%) risk of classical autoimmune (rheumatoid arthritis, multiple sclerosis, coeliac disease), autoinflammatory (osteoarthritis), and mixed-pattern (psoriasis) diseases. Osteoarthritis (genetic correlation (*r*_g_) = 0.28, *P* = 3.25 × 10^−15^), rheumatoid arthritis (*r*_g_ = 0.27, *P* = 1.5 × 10^−5^) and multiple sclerosis (*r*_g_ = 0.09, *P* = 4.00 × 10^−3^) were significantly genetically correlated with endometriosis. MR analysis suggested a causal association between endometriosis and rheumatoid arthritis (OR = 1.16, 95% CI = 1.02–1.33). eQTL analyses highlighted genes affected by shared risk variants, enriched for seven pathways across all four conditions, with three genetic loci shared between endometriosis and osteoarthritis (*BMPR2*/*2q33.1*, *BSN*/*3p21.31*, *MLLT10*/*10p12.31*) and one with rheumatoid arthritis (*XKR6*/*8p23.1*).

**LIMITATIONS, REASONS FOR CAUTION:**

We conducted the first female-specific GWAS analyses for immune conditions. Given the novelty of these analyses, the sample sizes from which results were derived were limited compared to sex-combined GWAS meta-analyses, which limited the power to use female-specific summary statistics to uncover the shared genetic basis with endometriosis in follow-up analyses. Secondly, the 39 genome-wide significant endometriosis-associated variants used as instrumental variables in the MR analysis explained approximately 5% of disease variation, which may account for the nominal or non-significant MR results.

**WIDER IMPLICATIONS OF THE FINDINGS:**

Endometriosis patients have a moderately increased risk for osteoarthritis, rheumatoid arthritis, and to a lesser extent, multiple sclerosis, due to underlying shared biological mechanisms. Clinical implications primarily involve the need for increased awareness and vigilance. The shared genetic basis opens up opportunities for developing new treatments or repurposing therapies across these conditions.

**STUDY FUNDING/COMPETING INTEREST(S):**

We thank all the UK Biobank and 23andMe participants. Part of this research was conducted using the UK Biobank Resource under Application Number 9637. N.R. was supported by a grant from the Wellbeing of Women UK (RG2031) and the EU Horizon 2020 funded project FEMaLe (101017562). A.P.M. was supported in part by Versus Arthritis (grant 21754). H.F. was supported by the National Natural Science Foundation of China (grant 32170663). N.R., S.A.M., and K.T.Z. were supported in part by a grant from CDMRP DoD PRMRP (W81XWH-20-PRMRP-IIRA). K.T.Z. and C.M.B. reported grants in 3 years prior, outside the submitted work, from Bayer AG, AbbVie Inc., Volition Rx, MDNA Life Sciences, PrecisionLife Ltd., and Roche Diagnostics Inc. S.A.M. reports grants in the 3 years prior, outside this submitted work, from AbbVie Inc. N.R. is a consultant for Endogene.bio, outside this submitted work. The other authors have no conflicts of interest to declare.

**TRIAL REGISTRATION NUMBER:**

N/A

## Introduction

Endometriosis is a chronic inflammatory condition that features the presence of endometrial-like tissue in locations outside the uterus, mainly in the pelvic cavity (Zondervan *et al.*, 2020). The most commonly accepted explanation for the origin of the majority of these endometrial deposits is retrograde menstruation, when menstrual blood containing endometrial cells travels up the fallopian tubes into the pelvic cavity (Sampson, 1927). However, this phenomenon is experienced by the majority of menstruating individuals (Halme *et al.*, 1984), leaving the question of why only some endometrial cells are able to adhere to peritoneal surfaces, thrive, and proliferate (Cramer and Missmer, 2002). Proliferation of the endometrial implants requires oestrogen, which is provided both systemically and also from localized production of aromatase (Zondervan *et al.*, 2020) and expression of oestrogen receptor beta (Bulun *et al.*, 2012), inhibition of TNF-α-induced apoptosis, increased interleukin-1β, which enhances cellular adhesion and proliferation, and localized inflammation (Han *et al.*, 2015). Endometriotic implants secrete various cytokines, chemokines, and prostaglandins, eliciting an inflammatory response that attracts macrophages, monocytes, neutrophils, T cells, and eosinophils. Impairment of the innate, and possibly adaptive, immune system in removing ectopic endometrial cells from the peritoneal cavity appears to play a role in endometriosis (Symons *et al.*, 2018; Zondervan *et al.*, 2020). In particular, altered function of natural killer cells and macrophages has been implicated, but it is unclear if these aberrations play a role in causation or are part of pathophysiology (Zondervan *et al.*, 2018, 2020).

Given the link with aberrant immune response, clinical and population-based studies have investigated if there is an association between endometriosis and autoimmune diseases, even postulating that endometriosis itself may be an autoimmune disorder (Gleicher *et al.*, 1987; Matarese *et al.*, 2003; Eisenberg *et al.*, 2012) because of the dysfunction in natural immunity. While auto-antibodies are not typically involved in the pathogenesis of endometriosis (Laudański *et al.*, 2023), and thus it is not classified as an autoimmune condition (https://www.autoimmuneinstitute.org/diseases_list/endometriosis/), a systematic review of published clinical and population studies suggested an increased risk of several autoimmune conditions (systemic lupus erythematosus, Sjögren’s syndrome, rheumatoid arthritis, autoimmune thyroid disorder, coeliac disease, multiple sclerosis, inflammatory bowel disease, and Addison’s disease) among females with endometriosis. However, most of the studies were limited by small sample sizes, selection biases, and lack of adjustment for confounding factors (Shigesi *et al.*, 2019), questioning the robustness of the evidence. In addition, none of the studies addressed whether there is a biological basis for any shared risk, information that is important for clinical translation.

Endometriosis and many immune-related conditions are common complex diseases, characterized by multifactorial aetiologies involving both genetic and environmental contributions. The cumulative effects of genetic risk variants result in an often considerable heritable component to disease risk (for endometriosis, estimated to be around 50% (Saha *et al.*, 2015), with 26% estimated to be due to common variants (Lee *et al.*, 2013)). Identifying these genetic variants, and particularly those shared between endometriosis and immune-related conditions, can provide valuable insights into their shared biological underpinnings. Genome-wide association studies (GWAS) are a powerful tool to uncover these associations by systematically scanning the genome for variants associated with a particular condition. By leveraging GWAS data, it is possible to explore how shared genetic risk factors contribute to the pathophysiology of these conditions, paving the way for a deeper understanding of their interconnected mechanisms and informing potential clinical diagnostic and therapeutic strategies.

Here we aim to investigate the association, and any shared biological basis, between endometriosis and 31 immunological disorders grouped into classical autoimmune, autoinflammatory, and mixed-pattern conditions (McGonagle and McDermott, 2006). We explore the association between endometriosis and immunological conditions in one of the largest available data sources, the UK Biobank (UKBB) data; conduct the first female-specific GWAS and the largest possible GWAS meta-analyses of conditions exhibiting significant phenotypic associations with endometriosis; use these datasets to investigate genetic correlations, potential causal pathways, and shared genetic risk variants between endometriosis and immunological diseases; and use endometrium and blood gene expression data to identify genes dysregulated by shared disease-associated variants (eQTL analyses).

## Materials and methods

### Phenotypic analysis study population and disease ascertainment

The UKBB is comprised of 500K individuals aged 40–69 at time of recruitment (2006–2010) from across the UK. The biobank was approved by the North West Multi-Centre Research Ethics Committee (MREC). In the UKBB, information was collected from participants during recruitment using questionnaires on socioeconomic status, behaviour, family history, and medical history. Participants were also followed up for cause-specific morbidity and mortality through linkage to disease registries, death registries, hospital admission records, and primary care data. In addition, a range of biological samples including blood, urine, and saliva, was collected from the participants. A more detailed description of the UKBB can be found in the UKBB protocol (Sudlow *et al.*, 2015).

Given that endometriosis is a gynaecological condition affecting those assigned female at birth, only individuals assigned female at birth (N = 273 404) were included in the phenotypic association analysis with the immunological conditions. From this point onwards we will refer to those assigned female at birth as females in this manuscript. Endometriosis was identified based on self-reported data from questionnaires and/or hospital records (ICD10/9: N801-809 and 617.1-9). A total of 31 immunological conditions were identified from self-reported data and/or hospital records (ICD10/9, see Supplementary Note) that were classified into three groups (94) as follows: (i) autoinflammatory conditions: acne, acute respiratory distress syndrome, erythema nodosum, giant cell/Takayasu arteritis, gout/pseudogout, total inflammatory bowel disease, Crohn’s disease, ulcerative colitis, osteoarthritis, sarcoidosis, (ii) classical autoimmune conditions: Addison’s disease, autoimmune gastritis, autoimmune thyroid disease, Graves’ disease, Hashimoto’s disease, coeliac disease, dermatomyositis/polymyositis, multiple sclerosis, myasthenia gravis, pemphigus/pemphigoid, primary biliary cirrhosis, rheumatoid arthritis, Sjögren's syndrome, systemic lupus erythematosus, systemic sclerosis, type 1 diabetes, vitiligo, (iii) Combination of autoinflammatory and autoimmune condition categories: ankylosing spondylitis, Behcet’s syndrome, reactive arthritis, psoriasis/psoriatic arthritis/psoriatic arthropathies. A common control set was defined as females without endometriosis diagnosis, excluding anyone with diagnoses of any of the 31 immunological conditions.

Potential confounding or mediating factors included age of recruitment, genetically determined ancestry, menopause status, age at menarche, parity, body size, BMI, and fat distribution (Rahmioglu *et al.*, 2015), alcohol consumption, smoking, infertility and diseases such as ovarian cancer (Kvaskoff *et al.*, 2015) and cardiovascular disease (Atsma *et al.*, 2006), which have been illustrated to be associated with endometriosis and some immunological conditions. Age at recruitment (which represents potential age-related cohort effects) and ancestry were considered *a-priori* variables to be included in the models. Many of the other factors were assessed only at baseline recruitment into UKBB, which for most females would have followed rather than coincided with, or preceded, an endometriosis diagnosis, and therefore the potential for confounding versus mediation effects could not be accurately assessed. Nevertheless, to assess their potential impact on the associations, factors associated both with endometriosis and immunological disease were included in a logistic regression model with endometriosis as exposure and immunological disease as outcome. None of these factors either showed >5% change in effect (potential confounders) or removal of effects (mediators), and therefore only *a-priori* variables age at recruitment and genetically determined ancestry were included in the models.

### Phenotypic association analysis

Phenotypic association analysis between endometriosis and immune conditions was conducted utilizing two different analysis methods: (i) a ‘gold standard’ cohort study design to incorporate temporality between diagnoses, where entry time was defined as the recruitment date into UKBB; (ii) a cross-sectional analysis to test for a simple association between risk of an immunological disease diagnosis with a diagnosis of endometriosis at any point in time, including all females in the UKBB. The cross-sectional analysis was supplemental to the cohort analysis and aimed at maximizing the power of association detection and investigation of the sensitivity of effect sizes to study design.

#### Cohort analyses

Cohort analyses were conducted for nine immunological diseases with a minimum of 1500 female cases to allow sufficient numbers of immunological disease cases after excluding prevalent immunological diseases diagnosed before cohort entry time or before the endometriosis diagnosis. The majority of females had immunological diseases diagnosed after endometriosis (66.8%, 1275 out of 1909 females with both diagnoses). Therefore, endometriosis was treated as the exposure, and immunological disease as the outcome, in the cohort analyses. This also fits with the observation that many individuals with endometriosis have symptom onset in their teens or twenties, often many years before their ultimate diagnosis (Surrey *et al.*, 2020). Females with an endometriosis diagnosis at the time of recruitment were classified as exposed, whereas those who had not had an endometriosis diagnosis at the time of recruitment were classified as unexposed. Those individuals who received an endometriosis diagnosis during follow-up, prior to any immunological disease diagnosis, contributed person-time to the unexposed group until the occurrence of endometriosis diagnosis, if any, and subsequently to the exposed group after diagnosis. For each immunological disease, females who had the respective immunological disease diagnosed before endometriosis or those who had the respective immunological disease diagnosed before cohort entry time or had immunological disease diagnosis time missing were excluded from cohort analysis (Table 1). In the cohort study, the risk of incident immunological diseases in females with and without endometriosis history was investigated using Cox proportional hazards regression models with calculated hazard ratios (HRs). The proportional hazards assumption was tested by function of ‘cox.zph’ in the ‘survival’ R (Version 3.6-4) library. In the cohort analysis, time to event was formulated from entry to the cohort until the end of follow-up time. The follow-up time (rather than age) is used as the underlying time variable, since the date of assessment is described in more detail with information on the exact date and months participants attended the assessment centre (to be used as the index date) in the UKBB. The end of follow-up time is the date of incident immunological diseases, death, loss to follow-up or end of follow-up (end date of follow-up is the date of last download of the dataset, which is 8 January 2019), whichever occurred first. Cohort analysis for each specific and categorized immunological disease was conducted with adjustment of age (categorical: <50, 50–60, ≥60) and genetically determined ancestry (categorical: white, non-white).

**Table 1.** Immunological disease risks among females with versus without endometriosis in UK Biobank utilizing cohort study design (N = 9 immunological disease with >1500 female cases; there was an insufficient number of cases to generate meaningful risk estimates for the 22 immunological conditions; see Supplementary Table S2 for results from a cross-sectional study design approach including 17 immunological conditions with >500 female cases).

**Immunological disease** ^a^	Total	Endo cases	Female controls	Follow-up time, years	HR (95% CI)	*P*-value	Person-years
**Overall (N=9 immunological disease)**	**14** **248**	**472**	**13** **776**	**9.43**	**1.31 (1.19**–**1.44)**	**<0.001**	**5750.90**
**Classic autoimmune disease (N=4)**	**2627**	**98**	**2529**	**9.78**	**1.42 (1.16**–**1.75)**	**<0.001**	**6982.99**
Multiple sclerosis	149	9	140	9.83	1.84 (0.93–3.61)	0.08	7309.16
Rheumatoid arthritis	1262	48	1214	9.81	1.57 (1.18–2.10)	0.002	7207.05
Coeliac disease	391	22	369	9.82	1.99 (1.30–3.07)	0.002	7299.37
Type 1 diabetes	511	17	494	9.82	1.38 (0.85–2.25)	0.19	7074.59
**Autoinflammatory disease (N=3)**	**13** **710**	**443**	**13** **267**	**9.46**	**1.28 (1.16**–**1.41)**	**<0.001**	**6080.81**
Ulcerative colitis	430	13	417	9.82	1.04 (0.58–1.84)	0.90	7052.21
Inflammatory bowel disease	316	12	304	9.82	1.40 (0.79–2.50)	0.25	4247.05
Osteoarthritis	13 371	416	12 955	9.48	1.31 (1.19–1.44)	<0.001	6196.13
**Mixed-pattern disease (N=2)**	**527**	**27**	**500**	**9.82**	**1.81 (1.21**–**2.71)**	**0.004**	**7237.373**
Ankylosing spondylitis	96	4	92	9.83	1.66 (0.61–4.52)	0.32	7332.22
Psoriasis	427	19	408	9.82	1.67 (1.05–2.65)	0.030	7254.40

Confounders included in the analyses are age at recruitment and genetically determined ancestry.

a For each immunological condition, females who had the respective immunological disease diagnosed before endometriosis or those who had the respective immunological disease diagnosed before cohort entry time or had immunological disease diagnosis time missing were excluded from cohort analysis. Here are the numbers of excluded individuals for each disease and disease category: overall immunological disease N = 50 6767, classical autoimmune disease N = 12 511, multiple sclerosis N = 1815, rheumatoid arthritis N = 4946, coeliac disease N = 2026, type 1 diabetes N = 1486, autoinflammatory disease N = 38 661, ulcerative colitis N = 2326, inflammatory bowel disease N = 115 404, osteoarthritis N = 34 568, mixed-pattern immunological disease N = 4240, ankylosing spondylitis N = 976, psoriasis N = 3662.

#### Cross-sectional analyses

Cross-sectional analyses were conducted for 17 immunological diseases that had at least 500 female cases in UKBB. A total of 14 immune conditions were excluded from analysis due to the number of cases being <500: reactive arthritis, Behcet’s syndrome, acute respiratory distress syndrome, erythema nodosum associated disease, pemphigus/pemphigoid, systemic sclerosis, vitiligo, primary biliary cirrhosis, Addison’s disease, myasthenia gravis, dermatomyositis polymyositis, Hashimoto’s disease, giant cell/Takayasu cell arteritis, and acne. In the cross-sectional study analysis, the prevalence of each specific and categorized immunological disease in females with and without a history of endometriosis diagnosis was investigated using logistic regression models with odds ratios (ORs) as risk measure. Cross-sectional study analysis for each specific and categorized immunological disease was conducted with adjustment of age and genetically determined ancestry.

All risk estimates were reported with 95% CIs and two-sided *P*-values. Person-years and mean follow-up time for each cohort analysis were calculated. All analyses were carried out using R software.

### GWAS and meta-analysis for immune conditions

With largest-scale GWAS meta-analysis results available for endometriosis (Rahmioglu *et al.*, 2023), the first step in genetic analysis was to conduct similar GWAS analyses for individual immune diseases in UKBB and meta-analyse these results, for each condition, with publicly available GWAS results. To avoid spurious associations due to ‘population stratification’ (confounding in association detection due to different proportions of individuals with different ancestries in case versus control groups), only genetically determined European ancestry individuals were included in the analysis. GWAS was conducted using UKBB data for females-only and sex-combined for eight immune conditions that showed significant phenotypic association with endometriosis: inflammatory bowel disease, osteoarthritis, ankylosing spondylitis, psoriasis, coeliac disease, multiple sclerosis, rheumatoid arthritis, and systemic lupus erythematosus. Controls were defined as a common control set without any diagnosis of immunological diseases or endometriosis within UKBB. The linear mixed model (LMM) implemented in BOLT (Version 2.4.1) (Loh *et al.*, 2015) was utilized for GWAS analysis to take into account relatedness in the data and to maximize the power of analysis. GWAS results were adjusted for a binary variable denoting the genotyping chip (the UKBB Axiom array or the UK BiLEVE array). Following standard protocols, single nucleotide polymorphisms (SNPs) included had a minimum minor allele frequency (MAF) of 1% and ≤60% missingness.

The largest published European ancestry GWAS results on the 8 selected immunological conditions were identified through literature (International Genetics of Ankylosing Spondylitis Consortium (IGAS) *et al.*, 2013; Okada *et al.*, 2014; de Lange *et al.*, 2017; International Multiple Sclerosis Genetics Consortium, 2019; Ricaño-Ponce *et al.*, 2020; Boer *et al.*, 2021; Wang *et al.*, 2021; Stuart *et al.*, 2022) and downloaded for meta-analysis with UKBB GWAS results. Following standard protocols (Anderson *et al.*, 2010), before meta-analysis, GWAS study-level quality controls (QC) were performed, and SNPs absent in the 1000 Genomes (Sung *et al.*, 2012) population-based reference panel with large missing value rates (≥60%) or lacking beta/odds ratio estimates in the publications were excluded. GWAS meta-analysis for each immunological disease was carried out using an inverse variance weighted fixed effect meta-analysis method implemented in METAL (Willer *et al.*, 2010). GWAS meta-analysis results were filtered, excluding SNPs with MAF < 1%, high heterogeneity (Het *I*^2^ > 90), and presence in <50% of effective sample size (N_eff_; N_eff_ = 4 NCases × NControls/(NCases + NControls)). All genetic analyses were conducted using the genome reference of the *Homo sapiens* (human) genome assembly GRCh37 (hg19). Genetic information on chromosome X was excluded. The major histocompatibility complex (MHC) region of Chr6:24000000–35000000 was excluded as it has a dense clustering of immune-relevant genes with complex (non-binary) polymorphisms unsuitable for use in GWAS analyses and very strong long-range linkage disequilibrium, which complicates the determination of the exact genes and alleles that are responsible for signals of disease association in the region (Trowsdale and Knight, 2013).

### Genetic correlation analysis

To identify shared genetic risk between endometriosis and different immunological conditions, we conducted genetic correlation analyses (Bulik-Sullivan *et al.*, 2015a; Bulik-Sullivan *et al.*, 2015b). For endometriosis, the GWAS meta-analysis results from the International Endometriosis Genome Consortium (IEGC) were used, including 52 350 cases and 504 157 controls from 20 GWAS studies excluding UKBB to prevent spurious associations due to overlapping study populations (Rahmioglu *et al.*, 2023). Genetic correlation analysis was conducted between endometriosis and immunological diseases via female-specific and sex-combined GWAS meta-analysis results via linkage disequilibrium score regression (LDSC) analysis (Bulik-Sullivan *et al.*, 2015a; Bulik-Sullivan *et al.*, 2015b). The LD score was calculated using software available at (http://github.com/bulik/ldsc), which was based on using standard protocols (Bulik-Sullivan *et al.*, 2015a; Bulik-Sullivan *et al.*, 2015b) on the 1000 Genomes European population. The significance threshold was set after Bonferroni correction as *P*-value = 0.00625 (0.05/8) to account for multiple testing of eight immunological diseases.

### MR analysis

These analyses use genetic variants as instrumental variables (IVs) to explore potential causal relationships between an exposure (endometriosis) and outcomes (the three immune-related diseases). For a detailed description of Mendelian randomization (MR) analyses using different methods and assumptions, we refer to Burgess *et al.* (2013). The potential causal relationships between endometriosis, as exposure, and immunological diseases that were genetically correlated, as outcome, were investigated by two-sample MR using the TwoSampleMR software (Hemani *et al.*, 2018). As IVs, we utilized the 39 established genome-wide significant LD-independent lead autosomal SNPs associated with endometriosis to assess whether endometriosis causally affects genetically correlated immune conditions (osteoarthritis, rheumatoid arthritis, or multiple sclerosis). Inverse variance weighted MR (MR‐IVW) was applied as the initial method to detect causal effects (Burgess *et al.*, 2013). As sensitivity analysis, other two‐sample MR methods, including weighted median MR (Bowden *et al.*, 2016) and MR‐Egger regression, were implemented in case the assumption of valid IVs was violated (Bowden *et al.*, 2015). While weighted median MR was shown previously to have lower Type 1 error rates than the inverse variance weighted method in a simulation analysis (Bowden *et al.*, 2016), MR‐Egger provides a method for sensitivity analysis to detect evidence of heterogeneity and pleiotropy of IVs, all factors that could influence the results (Bowden *et al.*, 2015). To detect IVs assuming heterogeneity and pleiotropy, MR PRESSO was applied to identify outliers (Verbanck *et al.*, 2018). Also, scatter plots (Bowden and Holmes, 2019) were generated to present the SNP–outcome association estimates versus the SNP–exposure associations in investigating the causal relationship using the MR models, including IVW, weighted median MR, and MR‐Egger regression (Verbanck *et al.*, 2018). These scatter plots illustrate the outlier IVs.

The strength of the 39 SNPs used in this analysis as IVs was evaluated by calculating *R*^2^ statistics using the ‘add_rsq()’ function in the TwoSampleMR software; the total *R*^2^ statistics for all 39 IVs was very low at 0.298%. F statistics were calculated for all 39 IVs (a sum of *Z*-statistics for each SNP squared) as 1656.30. Although the F statistic is relatively large for the 39 IVs, given a low *R*^2^ statistics for the 39 IVs used in the MR analysis, the set of IVs used for the MR analysis in this study was limited in power to assess causality between endometriosis and certain immunological diseases (Turley *et al.*, 2018).

### Multi-trait analysis of GWAS

Next we used multi-trait analysis of GWAS (MTAG) analysis to identify genetic variants contributing to the genetic correlations observed between endometriosis and immune conditions. By leveraging genetic correlations between conditions, MTAG boosts both the power of discovery for genetic variants associated with these individual conditions and the discovery of shared variants (Turley *et al.*, 2018). The input files for MTAG are the GWAS meta-analysis summary results files which were pre-processed by filtering out: (i) SNPs with MAF ≤1% or with a MAF difference ≥20% among datasets; (ii) restricting all analyses to a common set of SNPs present among datasets; (iii) multiple SNPs that were mapped to an identical chromosomal position among datasets; and (iv) SNPs with conflicting alleles due to strand alignment issues among datasets. *Z* scores (log(OR/SE)) were computed for all SNPs. After variant filtering, a total of 3 873 419 common SNPs between endometriosis, osteoarthritis, rheumatoid arthritis, and multiple sclerosis were included in the MTAG analysis. MTAG is a generalization of the standard inverse variance weighted meta-analysis framework (Turley *et al.*, 2018). Here endometriosis, osteoarthritis, rheumatoid arthritis, and multiple sclerosis pre-processed GWAS summary statistics were included in a single MTAG analysis.

In the results, MTAG outputs trait-specific effects estimated for each SNP, and the resulting *P*-value can be interpreted and used like those in single-trait GWAS (Turley *et al.*, 2018). For each disease, genome-wide significant lead SNPs were identified based on (i) achieving a genome-wide significant *P*-value (*P* < 5 × 10^−8^), (ii) being 500 kb distant from each other, and (iii) being independent (*r*^2^ < 0.1). Then, the genome-wide significant lead SNPs associated with respective diseases that are located within 1 Mb were identified, and the correlation (LD) between them was checked. Following common protocols, if the LD between lead SNPs of respective diseases was *r*^2^ ≥ 0.5, they were considered shared loci between those diseases.

### Functional annotation and pathway enrichments

To identify the genes regulated by the shared genetic variants, we have utilized eQTL maps from (i) the Genotype-Tissue Expression (GTEx) portal to identify whether they are eQTLs for genes across 49 human tissues from 838 donors with 15 201 samples (GTEx Consortium *et al.*, 2017) and (ii) eQTLGen to identify blood eQTLs from 31 684 individuals (Vosa *et al.*, 2021). Pathway enrichment analysis, testing whether the genes with expression perturbed by variants were significantly enriched for certain biological pathways, was conducted in Functional Mapping and Annotation of GWAS (FUMA) based on MTAG results for endometriosis, osteoarthritis, rheumatoid arthritis, and multiple sclerosis. Pathways included were limited to canonical pathways to limit multiple testing burden (Watanabe *et al.*, 2017).

### Data availability

The GWAS meta-analyses for immunological conditions made use of data from the UKBB (Application Number 9637) and publicly available GWAS summary statistics for immunological conditions (Okada *et al.*, 2014; de Lange *et al.*, 2017; International Multiple Sclerosis Genetics Consortium, 2019; Ricaño-Ponce *et al.*, 2020; Boer *et al.*, 2021; Wang *et al.*, 2021; Stuart *et al.*, 2022). GWAS data for endometriosis were based on the latest analyses of the IEGC (Rahmioglu *et al.*, 2023).

## Results

### Phenotypic association between endometriosis and immune conditions

The phenotypic association between endometriosis and immunological conditions was investigated in the UKBB using both retrospective cohort and cross-sectional study designs (see Materials and methods). Supplementary Table S1 shows factors that were determined as potential confounders or mediators in the association analyses between endometriosis and immunological diseases. Adding factors significantly associated with both endometriosis and immunological diseases in a logistic regression model with endometriosis as exposure and any immunological disease as the outcome (see Materials and methods), none were found to be confounders or mediators that significantly affected the effect size of association (>5% change). Genetically determined ancestry and age at recruitment were included *a priori* as potential confounders.

In both the retrospective cohort (Table 1) and cross-sectional analyses (Supplementary Table S2), females with endometriosis versus those without had a significantly increased risk for all immunological diseases combined (HR: 1.32 (1.20–1.45); OR: 1.32 (1.25–1.39)), classic autoimmune diseases (HR: 1.41 (1.15–1.74); OR: 1.24 (1.13–1.36)), autoinflammatory diseases (HR: 1.29 (1.17–1.43); OR: 1.33 (1.26–1.41)), and mixed-pattern diseases (HR: 1.88 (1.25–2.81); OR: 1.23 (1.10–1.52)).

Immunological diseases significantly associated with endometriosis in both analyses were: rheumatoid arthritis (OR: 1.22 (1.04–1.41), *P* = 0.011; HR: 1.57 (1.18–2.10), *P* = 0.002); coeliac disease (OR: 1.35 (1.06–1.70), *P* = 0.011; HR: 1.99 (1.30–3.07), *P* = 0.002); and osteoarthritis (OR: 1.35 (1.27–1.43), *P* < 0.001; HR: 1.31 (1.19–1.44), *P* < 0.001). In addition, in the ‘gold standard’ cohort analyses, psoriasis (HR: 1.67 (1.05–2.65), *P* = 0.030) was significantly associated with endometriosis. Two immunological conditions significantly associated with endometriosis in cross-sectional analysis, systemic lupus erythematosus (OR: 1.62 (1.14–2.24), *P* = 0.005) and gout (OR: 1.66 (1.18–2.26), *P* = 0.002), could not be tested in a cohort study design due to insufficient case numbers (Supplementary Table S2). Overall, females with endometriosis compared to females without known endometriosis exhibited a 14% increased risk for at least having one immunological disease (OR = 1.14 (1.08–1.21)), a 21% increased risk for at least having two immunological diseases (OR = 1.21 (1.05–1.39)), and a 30% increased risk for having at least three immunological diseases (OR = 1.30 (0.92–1.78)) at any point in their lifetime (*P* < 0.001) (Supplementary Table S3).

When stratifying by menopausal status, gynaecological surgery (hysterectomy/oophorectomy), or HRT use, effect sizes for the association between endometriosis and overall immunological disease risk remained largely unchanged (Supplementary Table S4).

### Genetic correlation between endometriosis and immunological diseases

We investigated whether the eight immunological diseases associated with endometriosis, either in cohort or cross-sectional analyses (ankylosing spondylitis, coeliac disease, inflammatory bowel disease, multiple sclerosis, osteoarthritis, psoriasis, rheumatoid arthritis, and systemic lupus erythematosus), shared a genetic basis with endometriosis through genetic correlation (*r*_g_) analyses (see Materials and methods). For this, we conducted GWAS focusing on female and combined-sex groups of European ancestry in the UKBB (Supplementary Table S5, see Materials and methods). To maximize statistical power, the combined-sex GWAS results from UKBB were meta-analysed with the largest available published GWAS summary statistics for each immune disease (Supplementary Figures S1, S2, S3, S4, S5, S6, S7 and S8).

Using these comprehensive GWAS meta-analyses, alongside the largest published endometriosis GWAS, we estimated the genetic correlations (*r*_g_) between endometriosis and these eight immunological diseases (see Materials and methods). Significant genetic sharing (*P* < 6.25 × 10^−3^) was observed for osteoarthritis (sex-combined *r*_g_ = 0.29, *P* = 3.25 × 10^−15^, female-specific *r*_g_ = 0.32, *P* = 1.76 × 10^−14^), rheumatoid arthritis (sex-combined *r*_g_ = 0.26, *P* = 1.54 × 10^−5^, female-specific *r*_g_ = 0.28, *P* = 0.001), and multiple sclerosis (sex-combined *r*_g_ = 0.09, *P* = 4.00 × 10^−3^, female-specific *r*_g_ = 0.25, *P* = 0.075) (Table 2). These results suggest a shared genetic basis between endometriosis and these conditions, potentially acting through common biological pathways.

**Table 2.** Genetic correlations from linkage disequilibrium score regression (LDSC) analysis between endometriosis and immunological disease.

	Female-only GWAS from UKBB				**Combined-sex GWAS meta-analysis** ^a^			
Immunological diseases	Sample size (cases:controls)	*H* ^2^ (SE)	*r*_g_ (SE)	*P*-value	Sample size	*H* ^2^ (SE)	*r*_g_ (SE)	*P*-value
Ankylosing spondylitis	547:162 403	0.006 (0.003)	0.308 (0.124)	0.013	1493:319 532	0.003 (0.001)	0.300 (0.124)	0.016
Coeliac disease	1706:162 403	0.013 (0.003)	0.215 (0.083)	0.010	7173:330 282	0.077 (0.005)	0.076 (0.054)	0.1619
Inflammatory bowel disease	2869:162 403	0.014 (0.003)	0.054 (0.085)	0.521	30 793:354 447	0.277 (0.028)	−0.041 (0.035)	0.237
Multiple sclerosis	1314:162 403	0.004 (0.003)	0.247 (0.139)	0.075	16 381:343 623	0.079 (0.005)	0.088 (0.031)	4.00 × 10^−3^
Osteoarthritis	39 866:162 403	0.051 (0.004)	0.322 (0.042)	1.76 × 10^−14^	77 052:378 169	0.046 (0.002)	0.278 (0.035)	3.25 × 10^−15^
Psoriasis	3036:162 403	0.012 (0.003)	0.043 (0.084)	0.605	22 558:347 726	0.239 (0.027)	0.066 (0.037)	0.076
Rheumatoid arthritis	4662:162 403	0.013 (0.003)	0.277 (0.079)	0.001	21 514:363 455	0.064 (0.009)	0.266 (0.062)	1.54 × 10^−5^
Systemic lupus erythematosus	545:162 403	0.003 (0.003)	0.154 (0.154)	0.316	6547:648 130	0.332 (0.048)	0.127 (0.071)	0.074

Multiple-testing correction for the number of diseases included in the analysis is applied (0.05/8 = 6.25 × 10^−3^) to determine significant correlations. UK Biobank (UKBB) endometriosis genome-wide association study (GWAS) results were excluded from endometriosis meta-analysis to avoid overlap in LDSC analysis with immunological conditions for which we have analysed this dataset. H^2^: heritability, *r*_g_: genetic correlation.

a Combined-sex UKBB GWAS was meta-analysed with published sex-combined GWAS if UKBB was not included in the published GWAS meta-analysis of these conditions. For breakdown of sample size, see Supplementary Table S5.

### Causal relationship between endometriosis and immunological diseases

To investigate whether endometriosis causally increases the risk of osteoarthritis, rheumatoid arthritis, and multiple sclerosis, MR analyses were conducted using 39 independent genetic variants associated with endometriosis (*P* < 5 × 10^−8^) as IVs. The primary results from the MR-IVW analysis (see Materials and methods) are shown in Table 3. Sensitivity analyses, including weighted median MR and MR-Egger regression, to validate the robustness of the results are shown in Supplementary Tables S6 and S7.

**Table 3.** Mendelian randomization (MR) results for endometriosis versus osteoarthritis, rheumatoid arthritis, and multiple sclerosis (results from sensitivity analyses are in Supplementary Table S6).

Immunological diseases	OR (95% CI)	*P*-value	**MR‐Egger heterogeneity** ^a^	**MR‐Egger pleiotropy** ^b^
**Osteoarthritis**				
Female-only UKBB (35 IVs^c^)	1.03 (0.98–1.09)	0.287	0.572	0.387
Combined-sex meta-analysis (35 IVs^c^)	1.01 (0.97–1.06)	0.495	0.159	0.800
**Rheumatoid arthritis**				
Female-only UKBB (39 IVs)	1.16 (1.02–1.33)	0.028	0.756	0.959
Combined-sex meta-analysis (36 IVs^c^)	1.06 (0.96–1.17)	0.220	0.611	0.435
**Multiple sclerosis**				
Female-only UKBB (39 IVs)	1.12 (0.87–1.43)	0.376	0.815	0.211
Combined-sex meta-analysis (38 IVs^c^)	1.04 (0.93–1.18)	0.484	0.063	0.202

a Test for heterogeneity *P*-value.

b Test for pleiotropy *P*-value.

c Outlier instrumental variables (IVs) were identified by MR PRESSO software (see Materials and methods and Supplementary Table S7).

For rheumatoid arthritis, the MR-IVW analysis suggested a potential causal relationship in females, with an odd ratio (OR) of 1.16 (95% CI: 1.02–1.33, *P* = 0.028). For osteoarthritis and multiple sclerosis, no significant causal relationship was identified in either combined-sex or female-only analyses (Table 3).

### Multi-trait analysis of endometriosis and immunological diseases: osteoarthritis, rheumatoid arthritis, and multiple sclerosis

To identify additional genetic variants associated with endometriosis, we employed MTAG (Turley *et al.*, 2018). This approach leverages shared genetic signals between endometriosis and conditions with which it showed shared genetic basis (osteoarthritis, rheumatoid arthritis, and multiple sclerosis) to enhance statistical power in detecting genetic associations. We conducted MTAG analysis for all four diseases simultaneously. Through this analysis, we identified 42 genome-wide significant (5 × 10^−8^) genetic variants significantly associated with endometriosis (Supplementary Table S8), 6 of which were not reported previously (Rahmioglu *et al.*, 2023): *ABHD1*/2p23.3, *TMEM131*/2q11.2, *XRCC4*/5q14.2, *PPP1R9A*/7q21.3, *XKR6*/8p23.1, and *TRPS1*/8p23.3 (Supplementary Fig. S9a–f). These 6 novel variants are linked to genes (eQTLs, see Materials and methods) with diverse biological roles: (i) *MSRA* and *PON2* protecting and repairing cells from oxidative stress in blood (Shin *et al.*, 2014; Manco *et al.*, 2021); (ii) *BLK* and *ZAP70* encoding enzymes that belong to tyrosine kinase family with roles in cell proliferation and differentiation in particular B-cell and T-cell development and adhesion (Wang *et al.*, 2010; Ichikawa-Tomikawa *et al.*, 2023); (iii) *ATRAID*, *SLC35F6*, *TMEM214*, and *XKR6* involved in apoptosis-related pathways (Li *et al.*, 2013; Stelzer *et al.*, 2016); and (iv) *TRPS1* encoding a transcription factor that represses GATA-regulated genes involved in progesterone resistance and endometriosis progression in the pelvis (Dyson *et al.*, 2014) (Supplementary Table S9).

In addition to endometriosis-specific results, MTAG analysis revealed 27 significant genetic variants for osteoarthritis (Supplementary Table S10), 28 for rheumatoid arthritis (Supplementary Table S11), and 64 for multiple sclerosis (Supplementary Table S12).

### Functional annotation of identified genome-wide significant variants and pathway analysis

**Figure 1.**
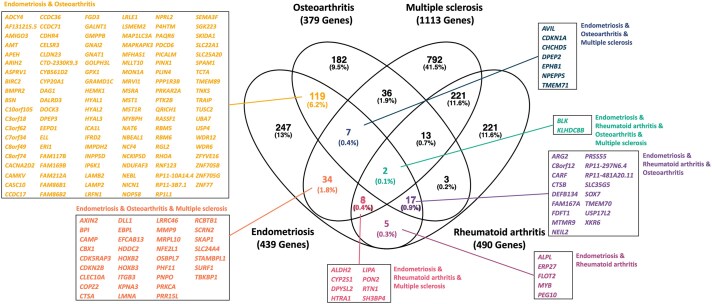
Overlap of genes associated with genome-wide association study (GWAS) lead single nucleotide polymorphisms (SNPs) in expression quantitative trait loci (eQTL) analyses for endometriosis (42 genome-wide significant lead SNPs are eQTLs for 439 genes), osteoarthritis (27 genome-wide significant lead SNPs are eQTLs for 379 genes), multiple sclerosis (64 genome-wide significant lead SNPs are eQTLs for 1113 genes), and rheumatoid arthritis (28 genome-wide significant lead SNPs are eQTLs for 490 genes) in various tissues in GTEx.

Functional analysis was performed to understand the biological roles of genetic variants identified in the MTAG analysis for endometriosis, osteoarthritis, rheumatoid arthritis, and multiple sclerosis. Using databases such as GTEx V8 (54 tissues) and eQTLGen (31 684 blood datasets), the genome-wide significant variants were mapped to genes associated with their expression (see Materials and methods). This analysis identified: 439 genes regulated by 42 endometriosis-associated variants, 379 genes regulated by 27 osteoarthritis-associated variants, 490 genes regulated by 28 rheumatoid arthritis-associated variants, and 1113 genes regulated by 64 multiple sclerosis-associated variants. Among the 439 genes linked to endometriosis variants, 192 were also regulated by a genetic variant associated with one or more of the other immune diseases, highlighting overlap in the genetic basis of these diseases. Figure 1 illustrates the shared genes regulated by variants associated with endometriosis and each of the three immune conditions: osteoarthritis, rheumatoid arthritis, and multiple sclerosis, respectively.

**Figure 2.**
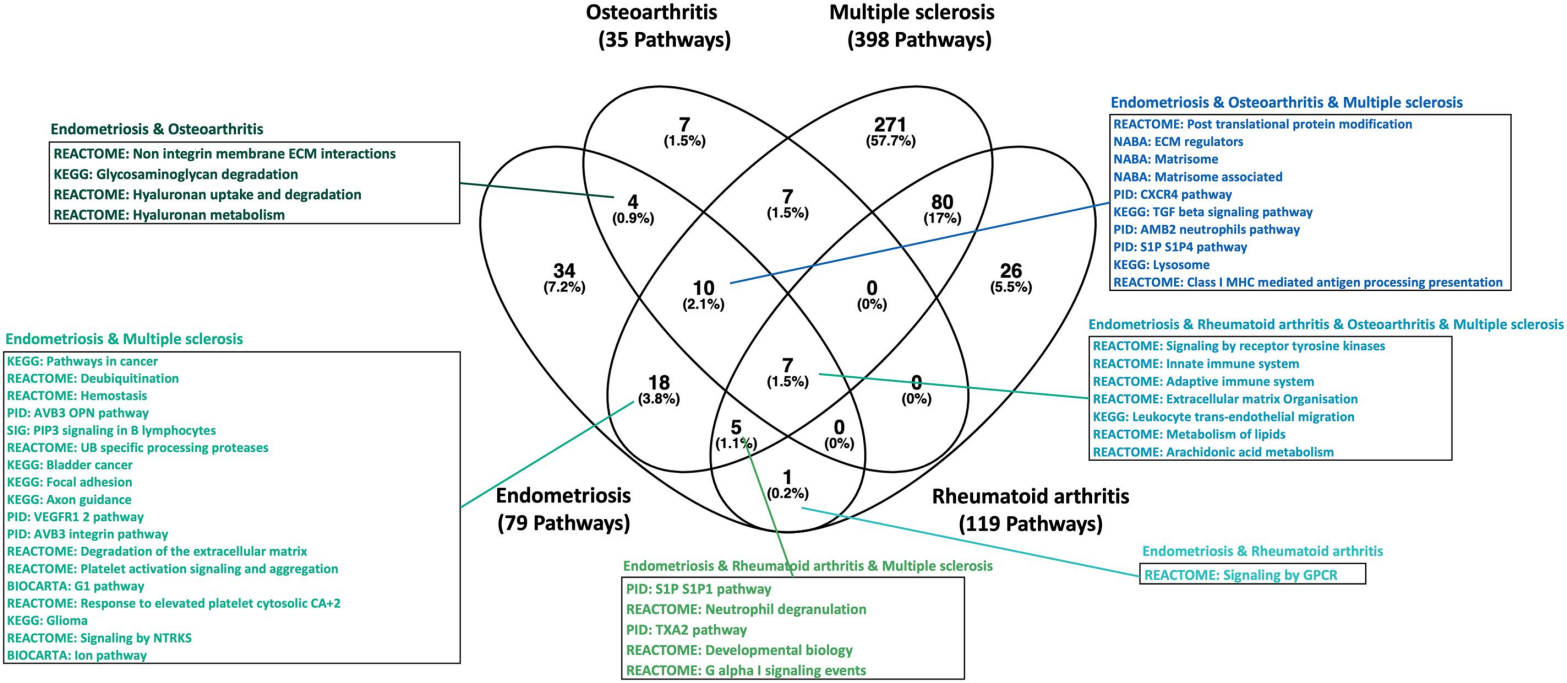
Overlap of pathways enriched with expression quantitative trait loci (eQTL) genes that are regulated by genome-wide association study (GWAS) lead single nucleotide polymorphisms (SNPs) associated with endometriosis, osteoarthritis, rheumatoid arthritis, and multiple sclerosis.

Pathway analysis (see Materials and methods) based on the identified genes per disease identified numerous canonical pathways enriched with these genes (Supplementary Tables S13, S14, S15 and S16). Investigating the overlap of enriched genetically driven pathways between endometriosis, osteoarthritis, multiple sclerosis, and rheumatoid arthritis, we discovered that 45 out of the 79 enriched pathways for endometriosis were also enriched in the other immune conditions (Fig. 2). In total, seven enriched pathways were shared across all four conditions, including ‘signalling by receptor tyrosine kinases’, ‘innate immune system’, ‘adaptive immune system’, ‘extracellular matrix organization’, ‘leukocyte trans-endothelial migration’, ‘lipid metabolism’, and ‘arachidonic acid metabolism’ (Supplementary Fig. S10). Within these overlapping enriched pathways, there are genes shared between conditions and also genes specific to each condition contributing to the pathway. For example, of the 21 genes enriched from endometriosis in ‘signalling by reception tyrosine kinase’, 8 are shared with osteoarthritis, including *NCF4*, *LAMB2*, *RHOA*, *MST1*, *MST1R*, *MAPKAPK3*, *DOCK3*, and *PTK2B*, and 3 are shared with multiple sclerosis, including *ITGB3*, *PRKCA*, and *MMP9* (Supplementary Fig. S10a).

A very interesting pathway, ‘extracellular matrix organization’, included many shared genes of potentially relevant biology: *MMP9*, *PRKCA*, and *ITGB3* (Supplementary Fig. S10d). *MMP9* encodes for a metalloproteinase that has a purported role in the progression of invasion in endometriosis as well as angiogenesis and fibrosis (Ke *et al.*, 2021), has involvement in a variety of inflammatory autoimmune diseases, and has been suggested to be a therapeutic target for autoimmune conditions (Ram *et al.*, 2006; Liu *et al.*, 2019). *PRKCA* is involved in immune cell trafficking, and *ITGB3* is coding for integrin β3 expression, which is associated with autoimmune conditions including multiple sclerosis (Du *et al.*, 2016).

Another enriched pathway across the four conditions is ‘arachidonic acid metabolism’; of the five endometriosis genes enriched in this pathway, four are shared with the other three immune conditions, namely, *DPEP3*, *GPX1*, *DPEP2*, and *PON2* (Supplementary Fig. S10g). Arachidonic acid-derived prostaglandins contribute to inflammation through their role as intercellular pro-inflammatory mediators and promote excitability of the peripheral somatosensory system, contributing to pain exacerbation (Jang *et al.*, 2020).

### Identification of shared genetic variants between endometriosis and immune diseases

A total of 12 genome-wide significant lead SNPs for osteoarthritis, rheumatoid arthritis, and multiple sclerosis were mapped within 1 Mb of endometriosis genome-wide significant lead SNPs, with four of them tagging the same genetic signal (*r*^2^ > 0.5) (Table 4, Supplementary Fig. S9a–f). Specifically, three SNPs were shared with osteoarthritis (*BMPR2*/2q33.1, *BSN*/3p21.31, and *MLLT10*/10p12.31), and one was shared with both osteoarthritis and rheumatoid arthritis (*XKR6*/8p23.1). MTAG association results of these 12 endometriosis SNPs in relation to osteoarthritis, rheumatoid arthritis, and multiple sclerosis are provided in Supplementary Table S17.

**Table 4.** Genome-wide significant lead single nucleotide polymorphisms (SNPs) associated with endometriosis (ENDO) and rheumatoid arthritis (RA), osteoarthritis (OA), or multiple sclerosis (MS) that are located within 1 Mb, with LD between them.

Locus	Traits	SNP	Chr	Position	EA (Frq)	OR (95% CI)	*P*-value	LD (r2)	**eQTLs results** *
GREB1/2p25.1	ENDO	rs12030576	1	115817221	G (0.65)	1.02 (1.01–1.03)	1.94 × 10^−14^	0.002	*TSPAN2*
	RA	rs6679677	1	114303808	C (0.10)	0.85 (0.84–0.87)	1.61 × 10^−92^		*AP4B1*, *APBA2*, *CD247*, *CD5*, *CD6*, *CTLA4*, *DCLRE1B*, *FBLN2*, *FOXP3*, *GZMB*, *IL10RA*, *IL2RA*, *MAF*, *MED15*, *PHTF1*, *RNF214*, *RTKN2*, *SLAMF1*, *ST8SIA1*, *STAP1*, *WLS*, *ZNF831*
DNM3/1q24.3	ENDO	rs2421985	1	172099136	T (0.52)	0.98 (0.97–0.98)	1.94 × 10^−14^	0.0001	*METTL13*
	OA	rs55698100	1	174227433	T (0.73)	1.01 (1.01–1.02)	7.97 × 10^−10^		*CACYBP*, *DARS2*, *GPR52*, *KIAA0040*, *KLHL20*, *MRPS14*, *PRDX6*, *RABGAP1L*, *RC3H1*, *SERPINC1*
ETAA1/2p14	ENDO	rs11126143	2	67865408	C (0.32)	1.02 (1.01–1.02)	6.08 × 10^−10^	0.01	None
	MS	rs12622670	2	68646536	C (0.46)	0.96 (0.98-0.99)	1.56 × 10^-9^		*CNRIP1*, *PLEK*, *PPP3R1*
BMPR2/2q33.1	ENDO	rs72928925	2	203554324	A (0.82)	1.02 (1.02–1.03)	9.61 × 10^−10^		*BMPR2*, *CARF*, *RAM117B*, *ICA1L*, *NBEAL1*
	OA	rs72928605	2	203832120	A (0.87)	1.02 (1.01–1.02)	4.33 × 10^−8^	0.53	*BMPR2*, *CARF*, *CYP20A1*, *FAM117B*, *ICA1L*, *NBEAL1*, *NOP58*
	RA	rs3087243	2	204738919	G (0.45)	1.03 (1.02–1.04)	2.39 × 10^−9^	0.001	*CTLA4*, *ICOS*
BSN/3p21.31	ENDO	rs6774202	3	49687779	T (0.17)	1.03 (1.01–1.03)	9.70 × 10^−12^	0.97	*AMT*, *ARIH2*, *APEH*, *C3orf62*, *CACNA2D2*, *CCDC36*, *CCDC71*, *CDHR4*, *DAG1*, *DALRD3*, *FAM212A*, *GMPPB*, *GPX1*, *HEMK1*, *HYAL3*, *IMPDH2*, *IP6K1*, *KLHDC88*, *MAPKAPK3*, *MON1A*, *MST1R*, *NAT6*, *NCKIPSD*, *NDUFAF3*, *NICN1*, *P4HTM*, *PRKAR2A*, *QRICH1*, *RBM6*, *RHOA*, *RNF123*, *TCTA*, *TRAIP*, *UBA7*, *WDR6*
	OA	rs6809879	3	49936102	G (0.18)	1.02 (1.01–1.02)	7.71 × 10^−9^		*AMT*, *APEH*, *ARIH2*, *BSN*, *CACNA2D2*, *CAMKV*, *CDHR4*, *CTD-2330K9.3*, *DOCK3*, *FAM212A*, *GMPPB*, *GPX1*, *HEMK1*, *HYAL3*, *IP6K1*, *KLHDC8B*, *MAPKAPK3*, *MON1A*, *MST1*, *MST1R*, *NAT6*, *NICN1*, *P4HTM*, *RASSF1*, *RBM5*, *RBM6*, *RHOA*, *RNF123*, *SEMA3F*, *TRAIP*, *UBA7*, *WDR6*
EBF1/5q33.3	ENDO	rs2964485	5	157904839	G (0.22)	0.98 (0.97–0.99)	1.11 × 10^−8^	0.001	None
	MS	rs2546890	5	158759900	A (0.52)	1.05 (1.04–1.07)	7.71 × 10^−13^		*UBLCP1*
PPP1R9A/7q21.3	ENDO	rs34751086	7	94592691	C (0.87)	1.02 (1.02–1.03)	3.23 × 10^−8^	0.03	*ASB4*, *CASD1*, *PEG10*, *PON1*, *PON2*, *PPP1R9A*
	OA	rs6966540	7	95727967	T (0.63)	0.99 (0.98–0.99)	1.46 × 10^−9^		*DYNC1I1*, *SLC25A13*
XKR6/8p23.1	ENDO	rs12542037	8	10758496	A (0.45)	1.02 (1.01–1.02)	7.02 × 10^−11^		*BLK*, *CTSB*, *FAM167A*, *FDFT1*, *MSRA*, *MTMR9*, *NEIL2*, *RP11-297N6*, *RP1L1*, *SLC35G5*, *XKR6*
	OA	rs4240673	8	10787612	T (0.45)	1.01 (1.01–1.02)	2.24 × 10^−12^	0.9	*BLK*, *CTSB*, *FAM167A*, *FDFT1*, *MSRA*, *MTMR9*, *NEIL2*, *RP11-297N6.4*, *RP1L1*, *SLC35G5*, *SOX7*, *XKR6*
	RA	rs2736340	8	11343973	C (0.26)	0.97 (0.96–0.98)	2.78 × 10^−9^	0.3	*ARG2*, *BLK*, *CTSB*, *FAM167A*, *FDFT1*, *GGA2*, *MTMR9*, *NEIL2*, *RP11-297N6*, *SLC35G5*, *XKR6*
MLLT10/10p12.31	ENDO	rs11012732	10	21830104	A (0.66)	0.98 (0.97–0.98)	3.84 × 10^−16^	0.7	*CASC10*, *MLLT10*, *NEBL*
	OA	rs12357321	10	21790476	G (0.68)	0.99 (0.98–0.99)	3.28 × 10^−9^		*CASC10*, *MLLY10*, *NEBL*
VEZT/12q22	ENDO	rs12320196	12	95645385	A (0.52)	0.98 (0.98–0.99)	5.37 × 10^−12^	0.003	*FGD6*, *NDUFA12*, *NR2C1*, *VEZT*
	OA	rs2171126	12	94167220	C (0.49)	0.99 (0.98–0.99)	2.88 × 10^−9^		*SOCS2*
DLEU1/13q14.2	ENDO	rs1028862	13	51055134	G (0.86)	0.98 (0.97–0.98)	9.63 × 10^−10^	0.0005	None
	MS	rs9591325	13	50811220	T (0.93)	1.09 (1.06–1.12)	1.59 × 10^−10^		*CORO1C*, *DLEU1*, *EBPL*, *KPNA3*, *PHF11*
SKAP1/17q21.32	ENDO	rs67446770	17	46235745	A (0.41)	0.98 (0.98–0.99)	8.37 × 10^−10^	0.002	*CBX1*, *CDK5RAP3*, *HOXB2*, *HOXB3*, *HOXB4*, *LRRC46*, *MRPL10*, *NFE2L1*, *SCRN2*, *SKAP1*, *SNX11*
	MS	rs11079784	17	45702280	T (0.49)	0.96 (0.94–0.97)	1.97 × 10^−10^		*COPZ2*, *EFCAB13*, *HOXB2*, *ITGB3*, *NPEPPS*, *SCRN2*, *SKAP1*, *TBKBP1*

* Full eQTL results from 54 GTEx tissues and eQTLGen blood tissue are provided in Supplementary Table S18. Chr: chromosome, AE: effective allele, Frq: effective allele frequency, LD: linkage disequilibrium, eQTL: expression quantitative trait loci.

At the *BMPR2*/*2q33.1* locus, the lead SNPs rs72928925 for endometriosis and rs72928605 for osteoarthritis are both regulating expression (eQTLs) of the *BMPR2* gene in blood and oesophageal muscularis (Supplementary Table S18). *BMPR2* encodes a member of the BMP receptor family of transmembrane serine/threonine kinases. The ligands of this receptor are members of the TGF-β superfamily. The TGF-β signalling pathway, involved in diverse cellular processes including cell proliferation, differentiation, apoptosis, and migration invasion, was also one of the pathways enriched with 10 eQTL genes regulated by endometriosis, osteoarthritis, and multiple sclerosis-associated variants (Fig. 2, Supplementary Tables S13, S14, S15 and S16).

At the *BSN*/*3p21.31* locus, the lead SNP rs6774202 associated with endometriosis and rs6809879 with osteoarthritis are both regulating expression (eQTLs) of a diverse set of overlapping genes (Table 4) that are part of pathways enriched between endometriosis and the other three immune conditions (Supplementary Tables S13, S14, S15 and S16). In particular, *RHOA* is part of the ‘leukocyte trans-endothelial migration’ pathway that was enriched across all four conditions. This pathway enables leukocytes to migrate from blood to tissues during inflammation and immune surveillance by binding to adhesion molecules and crossing the vascular endothelium (Muller, 2011). Another interesting eQTL gene within this locus is *HYAL3* involved in hyaluronan metabolism and glycosaminoglycan degradation. Hyaluronic acid, a key component of the extracellular matrix, plays a role in wound healing, tissue regeneration, and joint lubrication. It is used to relieve joint pain and promote healing and has been shown to reduce pro-inflammatory mediators and osteoarthritis pain (Migliore and Procopio, 2015; Kobayashi *et al.*, 2020). Recent studies suggest hyaluronic acid may reduce endometriosis lesion size in mice, though it may also promote inflammation acutely (Yu *et al.*, 2021), warranting further research into its therapeutic potential for endometriosis.

A third shared locus is *XKR6*/*8p23.1*, where the lead endometriosis SNP rs12542037 is in strong LD with the lead genome-wide significant osteoarthritis and rheumatoid arthritis SNPs (Table 4). This locus is involved in the regulation of multiple genes, namely *BLK*, *CTSB*, and *MTMP9*, which play roles in innate and adaptive immune system pathways (Milacic *et al.*, 2024). In addition, *FDFT1*, regulated by the correlated genetic risk variants, encodes for squalene synthase that is involved in cholesterol biosynthesis. It is also enriched in lipid rafts, which play an important part in many cellular processes, including signal transduction pathways, membrane trafficking, cytoskeletal organization, apoptosis, cell adhesions, and migration (Simons and Toomre, 2000). The ‘lipid metabolism pathway’ is also enriched with genes regulated by genetic variants in each of the investigated four conditions (Supplementary Tables S13, S14, S15 and S16, Fig. 2, and Supplementary Fig. S10f). In the context of inflammatory conditions, lipid metabolism has been suggested to harbour targets for reducing inflammation without the undesirable side effects of anti-inflammatory therapies (Robinson *et al.*, 2022).

The fourth locus previously implicated and described for endometriosis and osteoarthritis is *MLLT10*/10p12.31, which harbours genes such as *MLLT10* associated with pain perception and maintenance in multiple tissues (Rahmioglu *et al.*, 2023).

## Discussion

We conducted an unprecedentedly comprehensive investigation of the association between endometriosis and risk of a wide range of immune conditions, using a female-only study population from the large-scale UKBB dataset. Our findings reveal a significant increase in the risk of autoimmune and autoinflammatory diseases among endometriosis patients, particularly rheumatoid arthritis (HR: 1.57 (1.18–2.10), *P* = 0.002), coeliac disease (HR: 1.99 (1.30–3.07), *P* = 0.002), osteoarthritis (HR: 1.31 (1.19–1.44), *P* < 0.001), and psoriasis (HR: 1.67 (1.05–2.65), *P* = 0.030). The UKBB study population (aged 40–69 years) includes a relatively low proportion of diagnosed endometriosis cases (3% females), which is lower than the estimated population prevalence (up to 10% (Zondervan *et al.*, 2020)). As a result, the presence of undiagnosed cases may have diluted estimated effect sizes, potentially driving associations towards the null (Zondervan *et al.*, 2002; Shafrir *et al.*, 2018). Despite this, our results align with findings from previous case/control and cohort studies, which suggested significant associations between endometriosis and rheumatoid arthritis (Relative Risk (RR): 1.46 (0.70–3.03), coeliac disease (RR: 1.39 (1.14–1.70)), multiple sclerosis (OR: 7.1 (4.4–11.3)) (Shigesi *et al.*, 2019), and psoriasis (RR: 1.75 (1.10–2.78))(Harris *et al.*, 2022). For systemic lupus erythematosus, although our cohort analysis was limited by sample size, cross-sectional data showed a significant association (OR: 1.62 (1.14–2.24)), consistent with prior longitudinal studies reporting an increased risk (HR: 2.03 (1.17–3.51)) (Harris *et al.*, 2016). Furthermore, our findings indicate that endometriosis patients are at a significantly increased risk of suffering from multiple immunological diseases, a trend that was most pronounced for autoinflammatory conditions (one, OR: 1.15 (1.08–1.22); two, OR: 1.26 (0.94–1.64); three, OR: 3.75 (1.24–9.18), *P* < 0.001). This trend, previously observed in a cross-sectional analysis in an adolescent and early adulthood cohort (Shafrir *et al.*, 2021), is now expanded to a broader age range in our study.

Our results strongly suggest a biological basis for the epidemiological associations observed between endometriosis and a variety of immune conditions. The genetic correlation analysis demonstrated that genetic factors contribute to the association between endometriosis and the increased risk of rheumatoid arthritis (*r*_g_-female = 0.28, *P* = 1 × 10^−3^, *r*_g_-combined-sex = 0.27, *P* = 1.54 × 10^−5^), osteoarthritis (*r*_g_-female = 0.32, *P* = 1.76 × 10^−14^, *r*_g_-combined-sex = 0.28, *P* = 3.25 × 10^−15^), and to a lesser extent, multiple sclerosis (*r*_g_-female = 0.25, *P* = 0.075, *r*_g_-combined-sex = 0.09, *P* = 4.00 × 10^−3^). These correlations could arise from several mechanisms: (i) endometriosis may causally lead to the development of these conditions; (ii) both conditions may share a common genetic cause; or (iii) multiple shared causes could be at play, and the direction of effect between them can be complex (Kraft *et al.*, 2020). Genetic correlation between complex diseases is driven by polygenic genetic architectures with many causal SNPs of small effect that act cumulatively into aggregated effects, which is the case for endometriosis and the immune conditions studied here.

The MR analysis found limited evidence of causality (endometriosis directly causing an immune condition), with a suggestive causal effect of endometriosis on rheumatoid arthritis in females (OR = 1.16, 95% CI = 1.02–1.33, *P* = 0.028). A recently published MR-based study also illustrated a suggestive causal association between endometriosis and rheumatoid arthritis (OR = 1.005, 95 CI: 1.001–1.009, *P* = 0.014) (Tang *et al.*, 2024). However, the power of MR analysis depends on using genetic variants that strongly predict the exposure (endometriosis). Even genome-wide significant variants often offer only modest prediction of exposure as they explain only a small proportion of the heritable variation, as is the case for endometriosis (Burgess, 2014). In our analysis, the 39 endometriosis-associated variants explained 4.8% of medically/surgically confirmed and 5.01% of stage III/IV disease risk (10% of disease risk) (Rahmioglu *et al.*, 2023), limiting the power to detect causal relationships. Our MR instruments would have been weighted towards risk for stage III/IV disease, in particular ovarian endometrioma (Rahmioglu *et al.*, 2023). Previous studies associating risk of autoimmune and inflammatory conditions with endometriosis included predominantly stage I/II cases (Harris *et al.*, 2016; Shafrir *et al.*, 2021), although some of this evidence was based on adolescents who may have been genetically predisposed to develop stage III/IV disease later in life (Shafrir *et al.*, 2021).

A clear limitation in available data in our analyses was the lack of large, female-specific GWAS datasets for immune conditions, which restricted our ability to draw stronger conclusions about causal inferences in genetic overlaps. While we conducted female-specific GWAS analyses in the UKBB, the sample sizes were limited compared to sex-combined GWAS meta-analysis in the literature. This limitation is particularly relevant for immune conditions with higher prevalence in females. Sex-specific genetic signatures are well documented in conditions that show sex-based variability (Voskuhl, 2011), and female-specific GWAS for immune conditions could reveal stronger genetic correlations with endometriosis and increased opportunity for the discovery of shared genetic signals. Future studies with larger female-specific GWAS results for immune conditions are needed to better understand these relationships.

While power limitations hamper the interpretation of causal relationships between endometriosis and osteoarthritis, rheumatoid arthritis, or multiple sclerosis, results of the genetic correlation analyses highlight a shared genetic basis. Clinically, we recommend increased awareness of the risk of comorbidity in endometriosis patients and vigilance for early signs of these immune conditions. Understanding the basis of genetic sharing regardless of causality is important, as understanding of the shared biological mechanisms of pathogenesis and pathophysiology could open new avenues for treatment development. Leveraging the shared genetic basis between endometriosis, osteoarthritis, rheumatoid arthritis, and multiple sclerosis via MTAG analysis, we identified many shared genetic variants, effector genes, and pathways that could aid the discovery of novel treatment targets.

Future genetic comorbidity analyses should also explore results for different endometriosis subtypes. Recent GWAS analyses have suggested that ovarian endometriosis has a different genetic basis than peritoneal disease (Rahmioglu *et al.*, 2023), but the sample sizes for which summary statistics were generated did not allow for sufficiently powered inclusion in the present analyses. Similarly, future analyses should explore signals for different subtypes of immunological diseases such as osteoarthritis (Boer *et al.*, 2021), as well as specific immunophenotypes of rheumatoid arthritis (Kubo *et al.*, 2024), once larger GWAS datasets become available. Lastly, genetic analyses were limited to European ancestry individuals, and larger GWAS across more diverse ancestry groups for all diseases are needed to add translational value.

In conclusion, our results show that females with endometriosis are at a modestly increased risk of both autoimmune (42%) and autoinflammatory (28%) conditions, and that comorbidity with osteoarthritis, rheumatoid arthritis, and to a more limited extent multiple sclerosis, is biologically underpinned. Clinically, our results highlight the importance of awareness among treating physicians about the increased risk of such comorbidities. Early detection of immunological conditions in individuals with endometriosis—and vice versa—could improve patient outcomes. While current clinical action is limited to increased vigilance, our results offer a wide range of novel avenues and targets for exploring mechanisms and potential cross-condition treatment development or drug repurposing.

## Supplementary Material

deaf062_Supplementary_Figure_S1

deaf062_Supplementary_Figure_S2

deaf062_Supplementary_Figure_S3

deaf062_Supplementary_Figure_S4

deaf062_Supplementary_Figure_S5

deaf062_Supplementary_Figure_S6

deaf062_Supplementary_Figure_S7

deaf062_Supplementary_Figure_S8

deaf062_Supplementary_Figure_S9

deaf062_Supplementary_Figure_S10

deaf062_Supplementary_Table_S1

deaf062_Supplementary_Table_S2

deaf062_Supplementary_Table_S3

deaf062_Supplementary_Table_S4

deaf062_Supplementary_Table_S5

deaf062_Supplementary_Table_S6

deaf062_Supplementary_Table_S7

deaf062_Supplementary_Table_S8

deaf062_Supplementary_Table_S9

deaf062_Supplementary_Table_S10

deaf062_Supplementary_Table_S11

deaf062_Supplementary_Table_S12

deaf062_Supplementary_Table_S13

deaf062_Supplementary_Table_S14

deaf062_Supplementary_Table_S15

deaf062_Supplementary_Table_S16

deaf062_Supplementary_Table_S17

deaf062_Supplementary_Table_S18

## Data Availability

The GWAS meta-analysis summary statistics for eight immunological diseases (ankylosing spondylitis, coeliac disease, inflammatory bowel disease, multiple sclerosis, osteoarthritis, psoriasis, rheumatoid arthritis, and systemic lupus erythematosus), including sex-combined and UK Biobank female-only results, are available in the EBI GWAS Catalog (Study Accession GCST90558086, GCST90558087, GCST90558088, GCST90558089, GCST90558090, GCST90558091, GCST90558092, GCST90558093, GCST90558094, GCST90558095, GCST90558096, GCST90558097, GCST90558098, GCST90558099, GCST90558100, GCST90558101). The GWAS meta-analysis summary statistics for endometriosis excluding 23andMe data are available from the EBI GWAS Catalog (Study Accession GCST90205183); endometriosis GWAS summary statistics from 23andMe, Inc. were made available under a data use agreement that protects participant privacy. Please contact dataset-request@23andme. com or visit research.23andMe.com/collaborate for more information and to apply to access the data.
