# Supplementary material for: The phenotypic and genetic association between endometriosis and immunological diseases
Source: Hum Reprod. 2025 Apr 22;40(6):1195–209. doi: 10.1093/humrep/deaf062 (PMC12127507; doi:10.1093/humrep/deaf062)
Supplement: deaf062_Supplementary_Figure_S1 [file deaf062_supplementary_figure_s1.pdf]

OXFORD
human reproduction

Human Reproduction, 2025, 1–2
https://doi.org/10.1093/humrep/deaf062
Supplementary Data

(a) i. Female-only UKBB GWAS

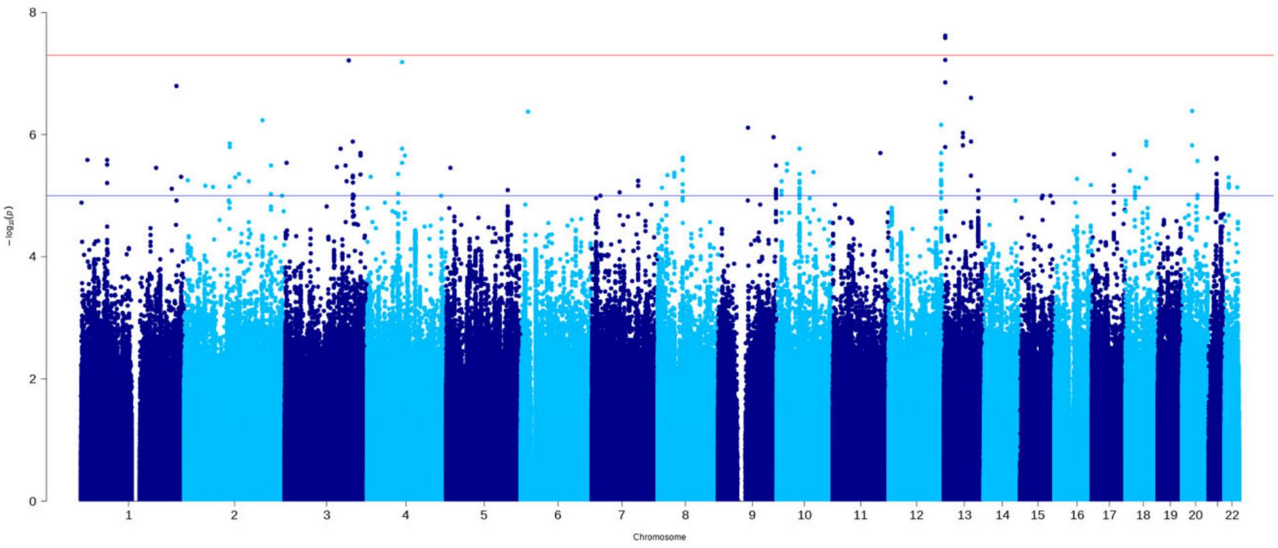

(a) ii. Sex-combined UKBB GWAS

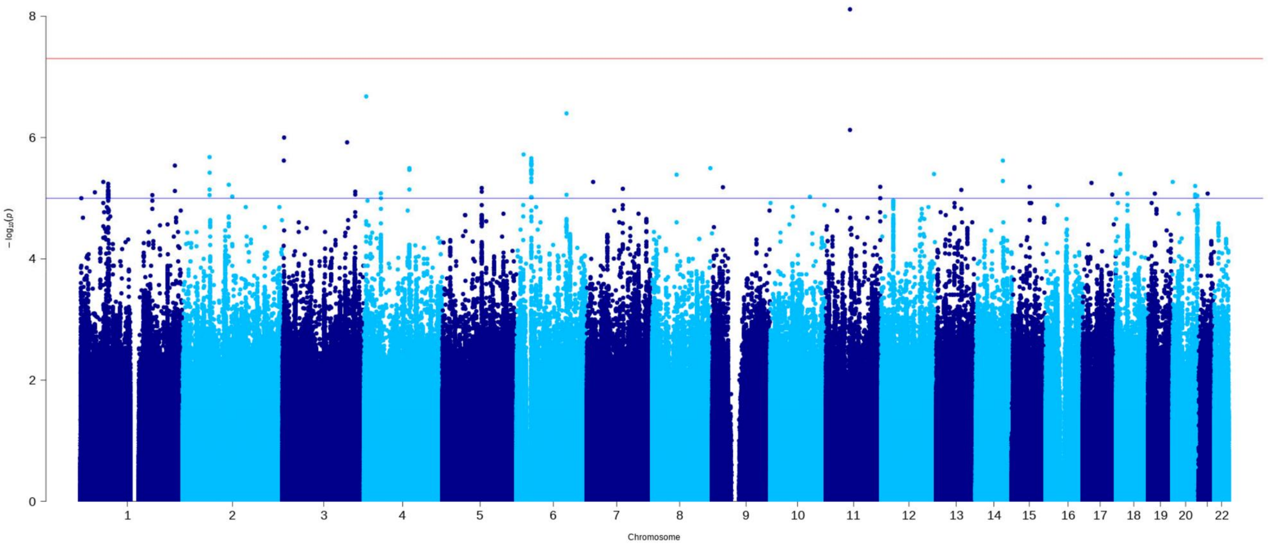

Supplementary Figure S1. Ankylosing spondylitis genome-wide association study (GWAS) results. (a) Manhattan plots and (b) Q–Q plots for i. Female-only and ii. Sex-combined GWAS summary results. In the Manhattan plots, *x*-axis is genomic location denoted by alternating coloured chromosomes. *Y*-axis is the negative log scale of the association *P*-value showing significance of association of each single nucleotide polymorphisms (SNPs) with ankylosing spondylitis. Each dot on the plot represents a SNP; red horizontal line represents genome-wide significance threshold ($P = 5 \times 10^{-8}$), and SNPs passing this threshold are significantly associated with ankylosing spondylitis; blue horizontal line represents the nominal significance threshold ($P = 5 \times 10^{-5}$).

(b) Q-Q plots for i. Female-only and ii. Sex-combined UKBB GWAS

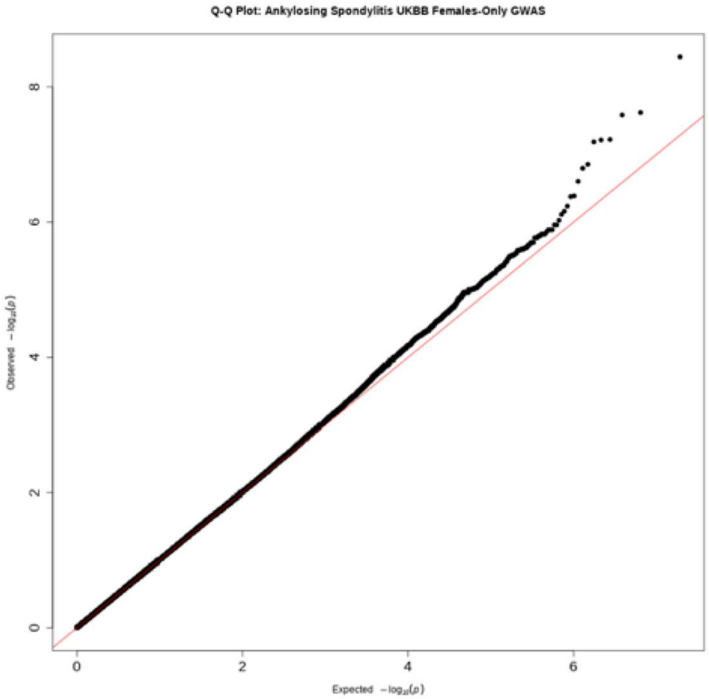

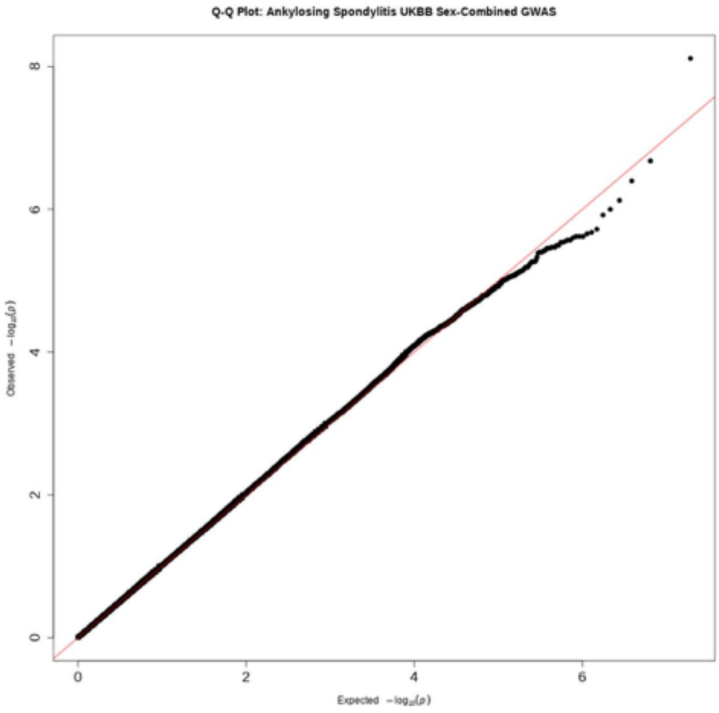

Supplementary Figure S1. Continued.
