# Supplementary material for: The phenotypic and genetic association between endometriosis and immunological diseases
Source: Hum Reprod. 2025 Apr 22;40(6):1195–209. doi: 10.1093/humrep/deaf062 (PMC12127507; doi:10.1093/humrep/deaf062)
Supplement: deaf062_Supplementary_Figure_S7 [file deaf062_supplementary_figure_s7.pdf]

OXFORD

human reproduction

Human Reproduction, 2025, 1–2

https://doi.org/10.1093/humrep/deaf062

Supplementary Data

(a) i. Female-only UKBB GWAS

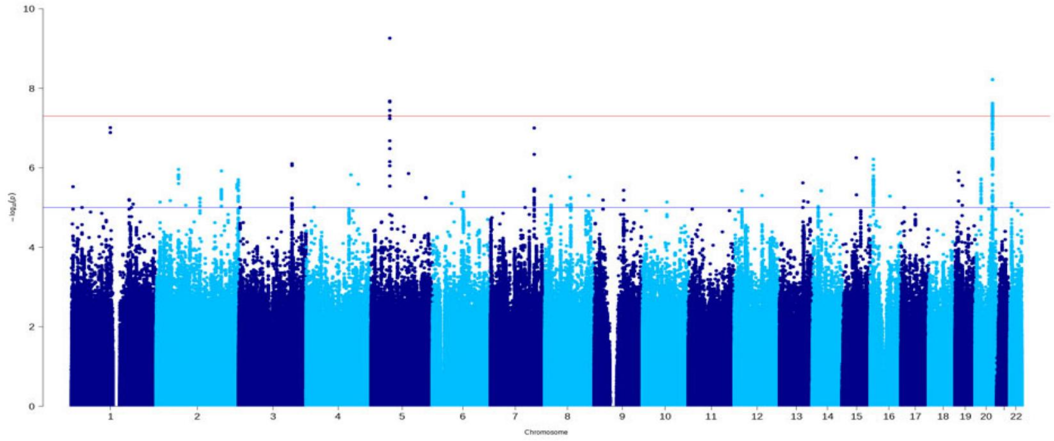

(a) ii. Sex-combined UKBB GWAS

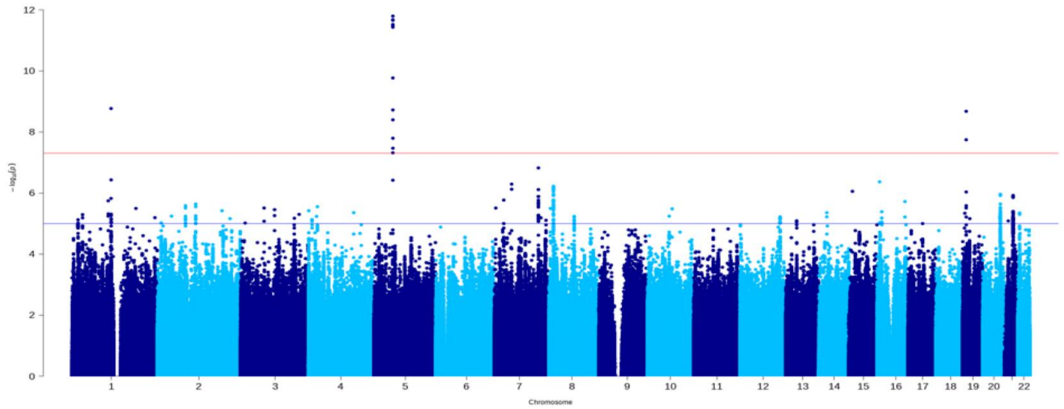

(a) iii. Meta-analysis of sex-combined GWAS results

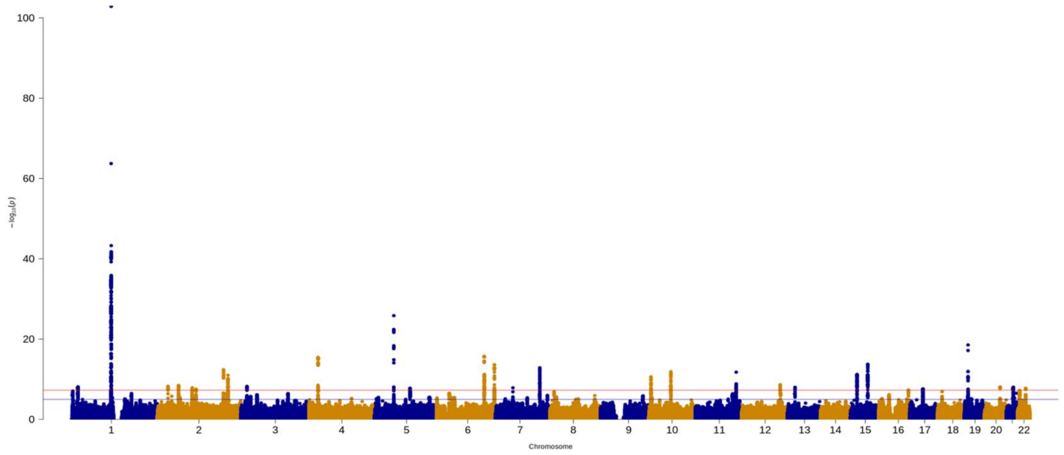

Supplementary Figure S7. Rheumatoid arthritis genome-wide association study (GWAS) results. (a) Manhattan plots, (b) Q–Q plots for i. Female-only, ii. Sex-combined GWAS, and iii. Meta-analysis of sex-combined summary results.

(b) Q-Q plots for i. Female-only, ii. Sex-combined iii. Meta-analysis of sex-combined GWAS.

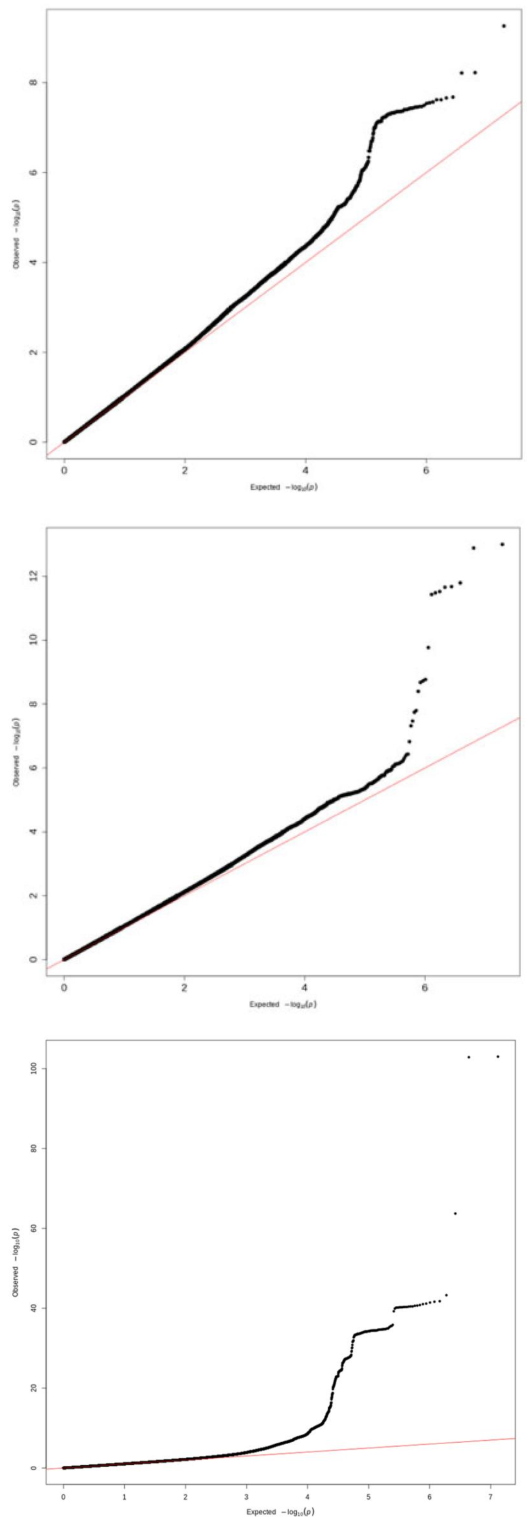

Supplementary Figure S7. Continued.
