# Supplementary material for: The phenotypic and genetic association between endometriosis and immunological diseases
Source: Hum Reprod. 2025 Apr 22;40(6):1195–209. doi: 10.1093/humrep/deaf062 (PMC12127507; doi:10.1093/humrep/deaf062)
Supplement: deaf062_Supplementary_Figure_S9 [file deaf062_supplementary_figure_s9.pdf]

OXFORD
human reproduction
Human Reproduction, 2025, 1–3
https://doi.org/10.1093/humrep/deaf062
Supplementary Data

(a) *ABHD1*/2p23.3

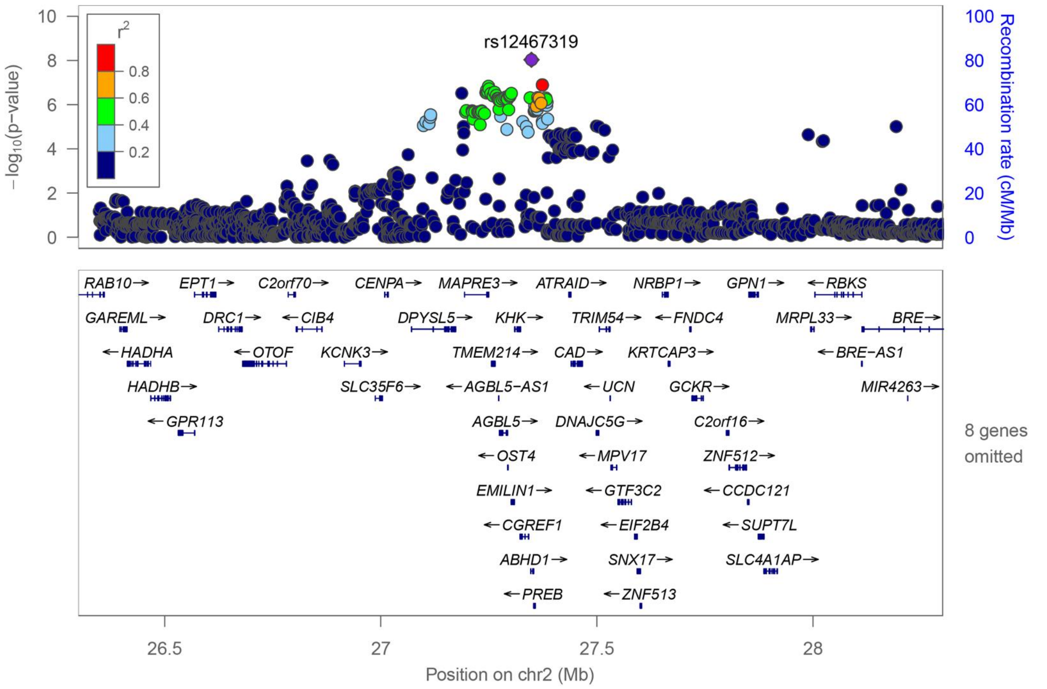

(b) *TMEM131*/2q11.2

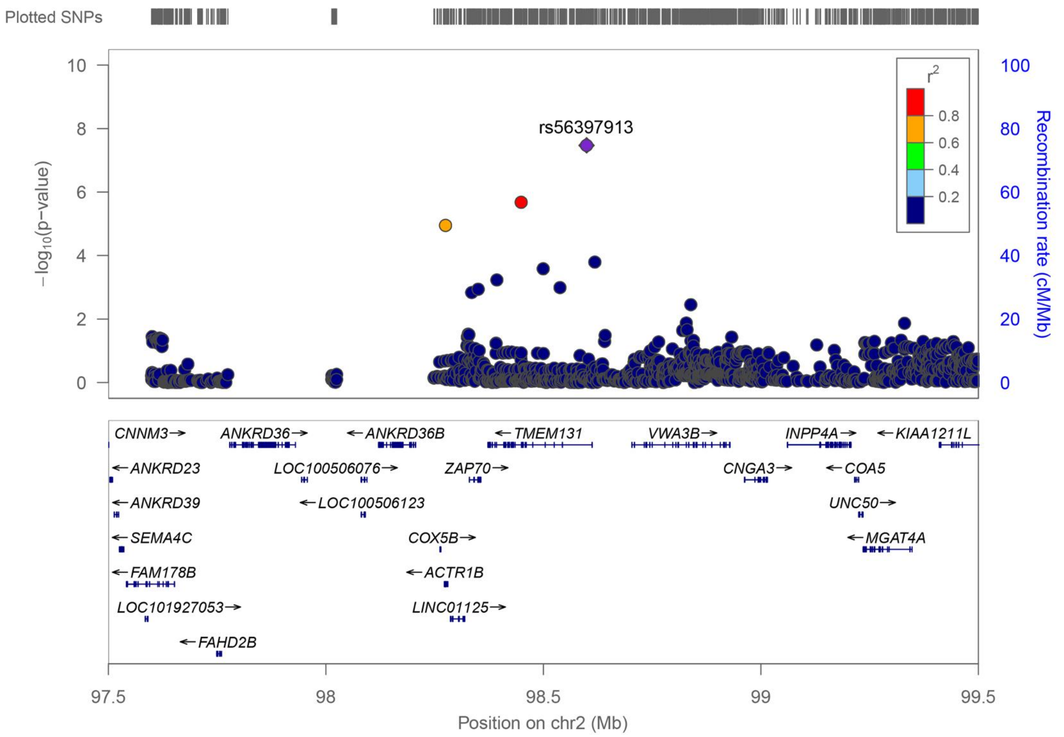

Supplementary Figure S9. Regional association plots for 6 novel genome-wide significant endometriosis loci from MTAG. (a) *ABHD1*/2p23.3, (b) *TMEM131*/2q11.2, (c) *XRCC4*/5q14.2, (d) *PPP1R9A*/7q21.3, (e) *XKR6*/8p23.1, (f) TRPS1/8p23.3. The association results are shown on the y-axis as –log10(*P*-value) and on the x-axis is the genomic location (hg 19). The top associated single nucleotide polymorphism (SNP) is coloured purple, and the other SNPs are coloured according to the strength of linkage disequilibrium (LD) with the top SNP by r2 in the European 1000 Genomes dataset.

(a) ***XRCC4*/5q14.2**

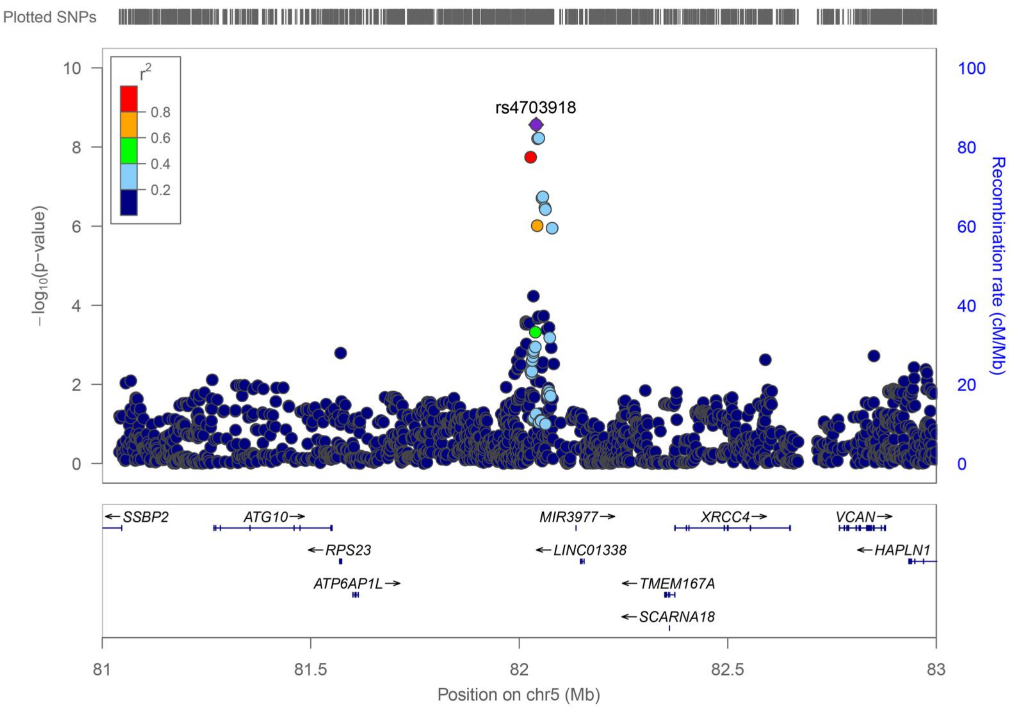

(b) ***PPP1R9A*/7q21.3**

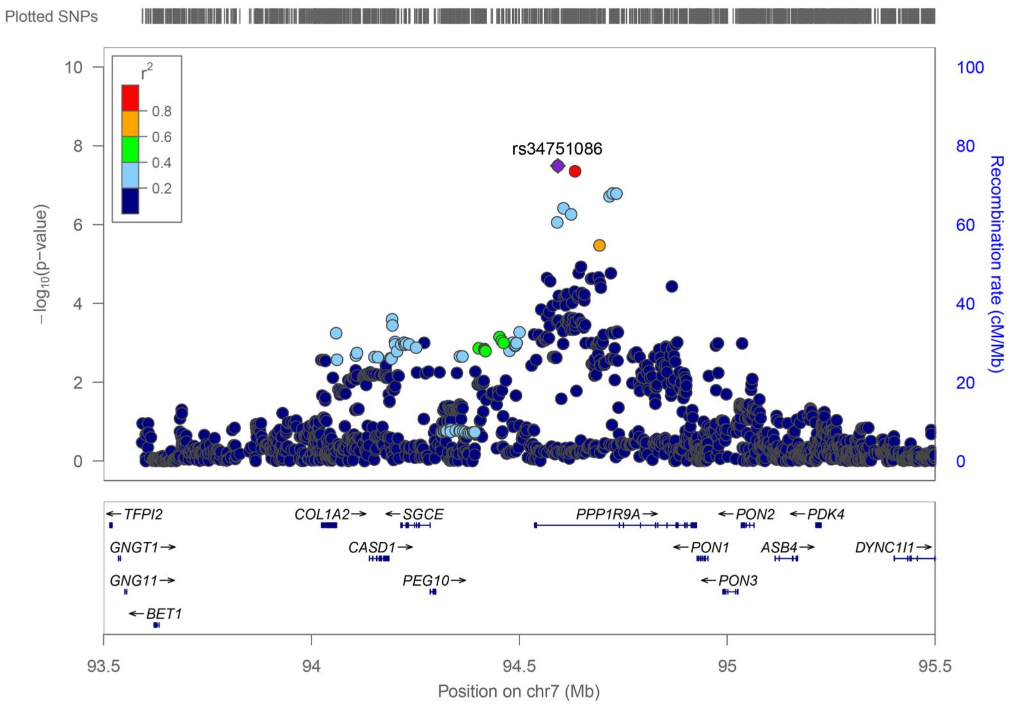

Supplementary Figure S9. Continued.

(c) *XKR6*/8p23.1

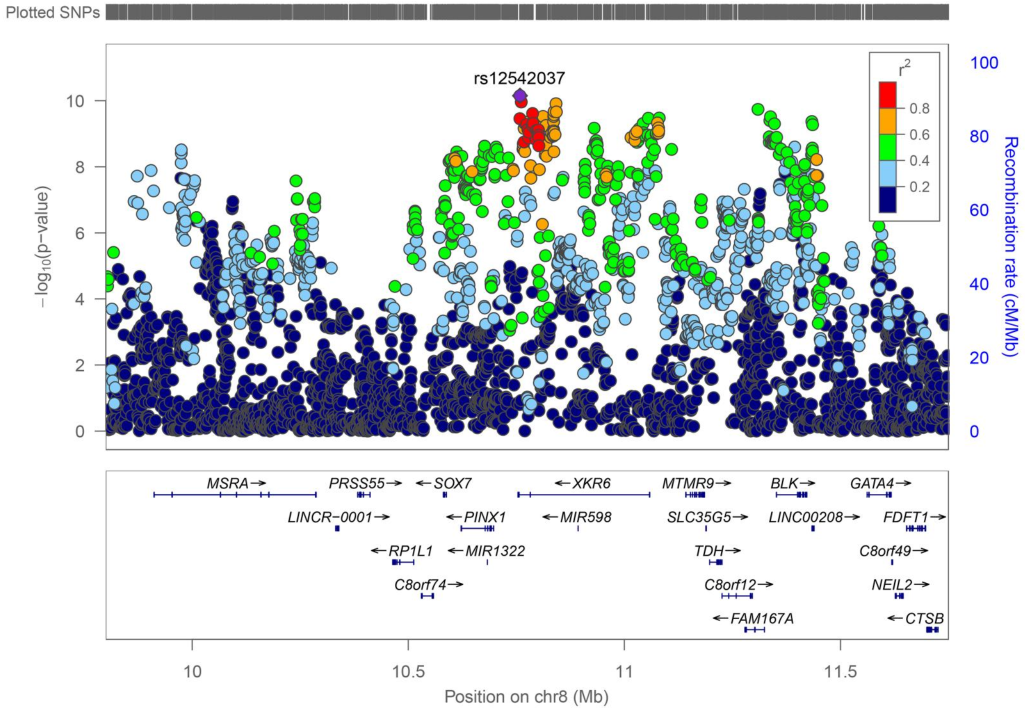

(f) *TRPS1*/8p23.3

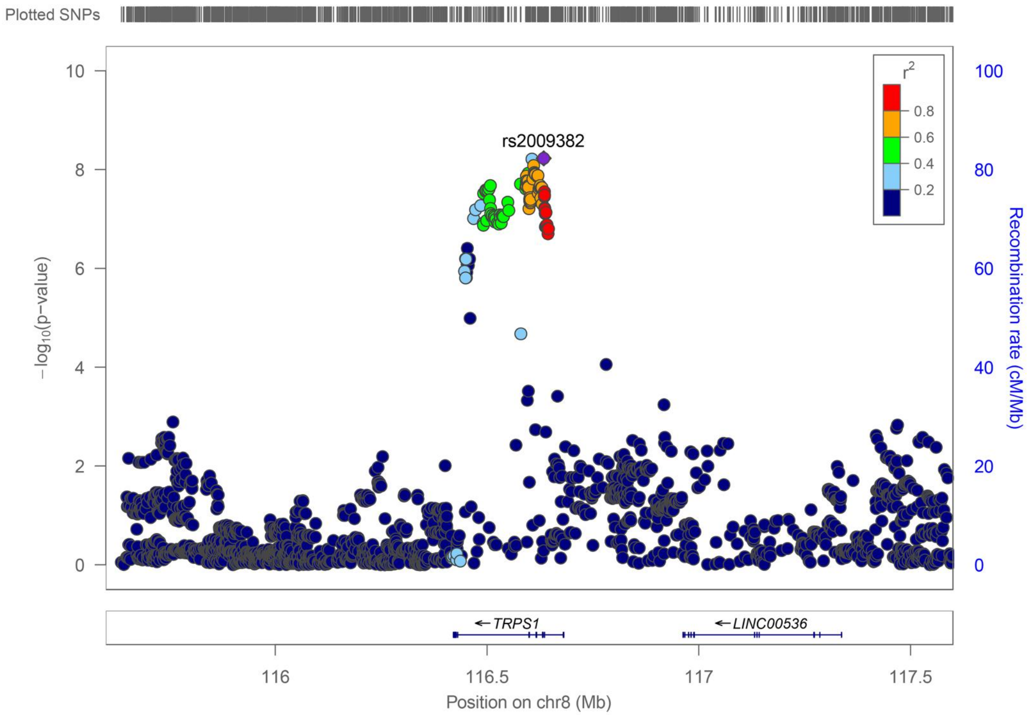

Supplementary Figure S9. Continued.
