# Supplementary material for: The phenotypic and genetic association between endometriosis and immunological diseases
Source: Hum Reprod. 2025 Apr 22;40(6):1195–209. doi: 10.1093/humrep/deaf062 (PMC12127507; doi:10.1093/humrep/deaf062)
Supplement: deaf062_Supplementary_Figure_S10 [file deaf062_supplementary_figure_s10.pdf]

*Human Reproduction*, 2025, 1–1
https://doi.org/10.1093/humrep/deaf062
Supplementary Data

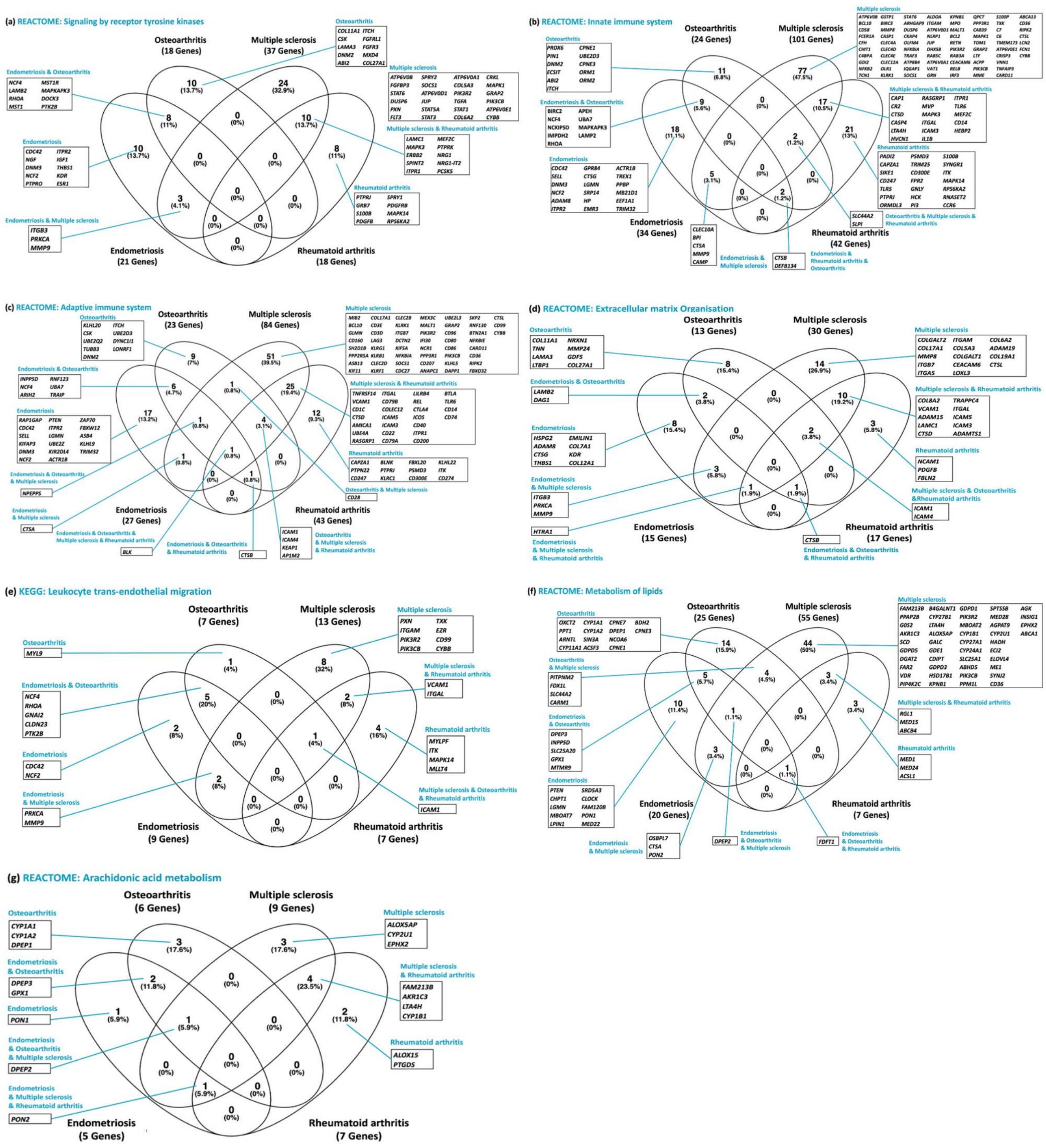


**Supplementary Figure S10. Top 7 pathways that are enriched with expression quantitative trait loci (eQTL) genes regulated by respective genome-wide significant lead single nucleotide polymorphisms (SNPs) across the four conditions: endometriosis, osteoarthritis, rheumatoid arthritis, and multiple sclerosis.** (a) REACTOME: signalling by receptor tyrosine kinases, (b) REACTOME: innate immune system, (c) REACTOME: adaptive immune system, (d) REACTOME: extracellular matrix organization, (e) KEGG: leukocyte trans-endothelial migration, (f) REACTOME: metabolism of lipids, (g) REACTOME: arachidonic acid metabolism.
