# Supplementary material for: The phenotypic and genetic association between endometriosis and immunological diseases
Source: Hum Reprod. 2025 Apr 22;40(6):1195–209. doi: 10.1093/humrep/deaf062 (PMC12127507; doi:10.1093/humrep/deaf062)
Supplement: deaf062_Supplementary_Table_S1 [file deaf062_supplementary_table_s1.pdf]

Human Reproduction, 2025, 1–1
https://doi.org/10.1093/humrep/deaf062
Supplementary Data

Supplementary Table S1. Characteristics of endometriosis cases versus controls and female immunological disease cases and controls in the UK Biobank data.

Characteristics	Endometriosis cases n = 8223	Female controls n = 265 181	Female immunological cases n = 64 620	Female controls n = 208 784
Age at recruitment [mean (SD)]	53.2 (7.9)	56.4 (8.0)	59.2 (7.1)	55.5 (8.0)
Ethnicity [n (%)]				
British European	6734 (81.9%)	214 652 (80.9%)	53 604 (83.0%)	167 782 (80.4%)
Others	1265 (15.4%)	42 070 (15.9%)	8 969 (13.9%)	34 366 (16.5%)
Had menopause [n (%)]	2829 (34.4%)	162 582 61.3%	44 594 (69.0%)	120 817 (57.9%)
Age at menarche [mean (SD)]	12.8 (1.7)	13.0 (1.6)	12.9 (1.7)	13.0 (1.6)
No. of live births [mean (SD)]	1.5 (1.2)	1.8 (1.2)	1.9 (1.2)	1.79 (1.19)
Ever taken HRT [n (%)]	4048 (49.2%)	99 873 (37.7%)	33 238 (51.4%)	70 683 (33.9%)
Ever taken oral contraceptive pills [n (%)]	7009 (85.2%)	213 444 (80.5%)	49 962 (77.3%)	170 491 (81.7%)
BMI in kg/m^2 [mean (SD)]	27.6 (5.4)	27.1 (5.2)	28.5 (5.8)	26.6 (4.9)
Waist–hip ratio [mean (SD)]	0.82 (0.07)	0.82 (0.07)	0.83 (0.07)	0.81 (0.07)
Body fat percentage [mean (SD)]	37.3 (6.8)	36.6 (6.9)	38.4 (6.9)	36.0 (6.8)
Body size at age 10 [n (%)]				
Thinner	2873 (34.9%)	82 543 (31.1%)	20 644 (31.9%)	64 772 (31.0%)
Average	3745 (45.5%)	131 602 (49.6%)	30 593 (47.3%)	104 754 (50.2%)
Plummer	1482 (18.0%)	46 075 (17.4%)	12 105 (18.7%)	35 452 (17.0%)
Smoking status [n (%)]				
Never smoker	4937 (60.0%)	157 129 (59.3%)	35 508 (54.9%)	126 558 (60.6%)
Ex-smoker	2420 (29.4%)	83 038 (31.3%)	22 538 (34.9%)	62 920 (30.1%)
Current smoker	828 (10.1%)	23 539 (8.9%)	6119 (9.5%)	18 248 (8.7%)
Alcohol drinking status [n (%)]				
Never drinker	422 (5.1%)	15 559 (5.9%)	4548 (7.0%)	11 433 (5.5%)
Past drinker	363 (4.4%)	9620 (3.6%)	3480 (5.4%)	6503 (3.1%)
Current drinker	7417 (90.2%)	239 193 (90.2%)	56 355 (87.2%)	190 255 (91.1%)
Infertility [n (%)]	442 (5.4%)	1970 (0.7%)	368 (0.6%)	2044 (1.0%)
History of ovarian cancer [n (%)]	105 (1.3%)	1129 (0.4%)	312 (0.5%)	922 (0.4%)
History of myocardial infarction [n (%)]	116 (1.4%)	4025 (1.5%)	1855 (2.9%)	2286 (1.1%)
History of angina [n (%)]	303 (3.7%)	9555 (3.6%)	4513 (7.0%)	5345 (2.6%)
Ever had coronary artery bypass graft [n (%)]	23 (0.3%)	756 (0.3%)	351 (0.5%)	428 (0.2%)
