# Supplementary material for: The phenotypic and genetic association between endometriosis and immunological diseases
Source: Hum Reprod. 2025 Apr 22;40(6):1195–209. doi: 10.1093/humrep/deaf062 (PMC12127507; doi:10.1093/humrep/deaf062)
Supplement: deaf062_Supplementary_Table_S2 [file deaf062_supplementary_table_s2.pdf]

OXFORD

human reproduction

Human Reproduction, 2025, 1–1
https://doi.org/10.1093/humrep/deaf062
Supplementary Data

Supplementary Table S2. Immunological disease risks among females with versus without endometriosis in UK Biobank utilizing cross-sectional study design (N = 17 immunological diseases with >500 female cases).

Immunological disease	Total females	Endometriosis cases (N = 8223)	Female controls (N = 265 181)	OR (95% CI)	P-value
**Overall (N=17 Immunological diseases)**	**64620 (23.64%)**	**2064 (25.10%)**	**62556 (23.59%)**	**1.32 (1.25–1.39)**	**<0.001**
**Classic autoimmune disease (N=9)**	**14764 (5.40%)**	**505 (6.14%)**	**14259 (5.38%)**	**1.24 (1.13–1.36)**	**<0.001**
Systemic lupus erythematosus	780 (0.29%)	36 (0.44%)	744 (0.28%)	1.62 (1.14–2.24)	0.005
Sjogren’s syndrome	803 (0.29%)	23 (0.28%)	780 (0.29%)	1.07 (0.68–1.59)	0.76
Multiple sclerosis	1571 (0.57%)	61 (0.74%)	1510 (0.57%)	1.23 (0.93–1.58)	0.13
Rheumatoid arthritis	5818 (2.13%)	187 (2.27%)	5631 (2.12%)	1.22 (1.04–1.41)	0.011
Coeliac disease	2023 (0.74%)	76 (0.92%)	1947 (0.73%)	1.35 (1.06–1.70)	0.011
Type 1 diabetes	1603 (0.59%)	57 (0.69%)	1546 (0.58%)	1.26 (0.95–1.64)	0.09
Autoimmune gastritis	1466 (0.54%)	48 (0.58%)	1418 (0.53%)	1.20 (0.88–1.58)	0.22
Graves’ disease	545 (0.20%)	23 (0.28%)	522 (0.20%)	1.28 (0.80–1.93)	0.27
Autoimmune thyroid disease	788 (0.29%)	29 (0.35%)	759 (0.29%)	1.17 (0.79–1.69)	0.42
**Autoinflammatory disease (N=6)**	**52027 (19.03%)**	**1638 (19.92%)**	**50389 (19.00%)**	**1.33 (1.26–1.41)**	**<0.001**
Crohn's disease	1382 (0.51%)	48 (0.58%)	1334 (0.50%)	1.18 (0.87–1.58)	0.27
Ulcerative colitis	2365 (0.87%)	82 (1.00%)	2283 (0.86%)	1.24 (0.98–1.55)	0.06
Inflammatory bowel disease	3527 (1.29%)	129 (1.57%)	3398 (1.28%)	1.28 (1.06–1.53)	0.009
Osteoarthritis	47642 (17.43%)	1493 (18.16%)	46149 (17.40%)	1.35 (1.27–1.43)	<0.001
Sarcoidosis	741 (0.27%)	26 (0.32%)	715 (0.27%)	1.39 (0.91–2.01)	0.1
Gout, pseudogout, crystal arthropathy	1117 (0.41%)	41 (0.50%)	1076 (0.41%)	1.66 (1.18–2.26)	0.002
**Mixed-pattern disease (N=2)**	**4379 (1.60%)**	**156 (1.90%)**	**4223 (1.59%)**	**1.23 (1.10–1.52)**	**0.002**
Ankylosing spondylitis	676 (0.25%)	28 (0.34%)	648 (0.24%)	1.59 (1.06–2.30)	0.019
Psoriasis	3697 (1.36%)	121 (1.47%)	3576 (1.35%)	1.18 (0.97–1.41)	0.08

Confounders included in the analyses are age at recruitment and genetically determined ancestry.
