# Supplementary material for: The phenotypic and genetic association between endometriosis and immunological diseases
Source: Hum Reprod. 2025 Apr 22;40(6):1195–209. doi: 10.1093/humrep/deaf062 (PMC12127507; doi:10.1093/humrep/deaf062)
Supplement: deaf062_Supplementary_Table_S3 [file deaf062_supplementary_table_s3.pdf]

Human Reproduction, 2025, 1–1
https://doi.org/10.1093/humrep/deaf062
Supplementary Data

Supplementary Table S3. Risks of multiple immunological diseases diagnosed in women with endometriosis.

	Immunological diseases overall		Autoimmune diseases		Autoinflammatory diseases		Mixed-pattern diseases	
**No. of diseases**	**Cases versus (vs) Controls**	**OR (95% CI)**	**Cases versus (vs) Controls**	**OR (95% CI)**	**Cases versus (vs) Controls**	**OR (95% CI)**	**Cases versus (vs) Controls**	**OR (95% CI)**
**One**	1762 vs 54 547	1.14 (1.08–1.21)	458 vs 13 115	1.11 (1.00–1.23)	1569 vs 48 694	1.15 (1.08–1.22)	156 vs 4198	1.16 (0.97–1.36)
**Two**	254 vs 6863	1.21 (1.05–1.39)	37 vs 1025	1.12 (0.77–1.58)	64 vs 1656	1.26 (0.94–1.64)	—	—
**Three**	48 vs 1146	1.3 (0.92–1.78)	10 vs 119	2.2 (0.98–4.26)	5 vs 39	3.75 (1.24–9.18)	—	—
**P-value (trend)**	$P < 0.001$		$P = 0.037$		$P < 0.001$		—	—
