# Supplementary material for: The phenotypic and genetic association between endometriosis and immunological diseases
Source: Hum Reprod. 2025 Apr 22;40(6):1195–209. doi: 10.1093/humrep/deaf062 (PMC12127507; doi:10.1093/humrep/deaf062)
Supplement: deaf062_Supplementary_Table_S4 [file deaf062_supplementary_table_s4.pdf]

Human Reproduction, 2025, 1–1
https://doi.org/10.1093/humrep/deaf062
Supplementary Data

Supplementary Table S4. Immunological disease risk among women with and without endometriosis stratified by menopause status and HRT use.

All immune diseases	Endometriosis OR (95% CI)*
**All women (N = 64 620: 2064 versus 62 556)**	1.32 (1.25–1.39)
**Had menopause (N = 44 594: 844 versus 43 750)**	1.26 (1.16–1.37)
**Pre-menopause (N = 17 707: 1138 versus 16 569)**	1.30 (1.21–1.39)
**Had HRT since menopause (N = 24 621: 553 versus 24 068)**	1.19 (1.07–1.32)
**Without HRT since menopause (N = 19 973: 291 versus 19 682)**	1.23 (1.07–1.41)
**Surgical menopause (N = 800: 76 versus 724)**	1.14 (0.84–1.56)
**Non-surgical menopause (N = 42 156: 660 versus 41 496)**	1.23 (1.12–1.34)

* Adjusted confounders include ethnicity and age at recruitment.
