# Supplementary material for: The phenotypic and genetic association between endometriosis and immunological diseases
Source: Hum Reprod. 2025 Apr 22;40(6):1195–209. doi: 10.1093/humrep/deaf062 (PMC12127507; doi:10.1093/humrep/deaf062)
Supplement: deaf062_Supplementary_Table_S5 [file deaf062_supplementary_table_s5.pdf]

OXFORD
human reproduction
Human Reproduction, 2025, 1–1
https://doi.org/10.1093/humrep/deaf062
Supplementary Data

Supplementary Table S5. Number of cases and controls of UK Biobank (UKBB)-based female-only and sex-combined genome-wide association studies (GWAS) for immunological conditions and summary of their meta-analysis with the largest publicly available European ancestry GWAS results.

	UKBB GWAS (cases:controls)			
**Immunological diseases**	**Female-only**	**Sex-combined**	**Published sex-combined GWAS (cases:controls)***	**Final sex-combined GWAS meta-analysis (cases:controls)**
Ankylosing spondylitis	547:162 403	1493:319 532	N/A	1493:319 532
Coeliac disease	1706:162 403	2640:319 532	4533:10 750	7173:330 282
Inflammatory bowel disease	2869:162 403	5751:319 532	25 042:34 915	30 793:354 447
Multiple sclerosis	1314:162 403	1883:319 532	14 498:24 091	16 381:343 623
Osteoarthritis	39 866:162 403	68 878:319 532	77 052:378 169	77 052:378 169**
Psoriasis	3036:162 403	6591:319 532	15 967:28 194	22 558:347 726
Rheumatoid arthritis	4662:162 403	7153:319 532	14 361:43 923	21 514:363 455
Systemic lupus erythematosus	545:162 403	673:319 532	5874:328 598	6547:648 130

* See studies by Boer *et al.* (2021), de Lange *et al.* (2017), International Genetics of Ankylosing Spondylitis Consortium (IGAS) *et al.* (2013), International Multiple Sclerosis Genetics Consortium (2019), Okada *et al.* (2014), Ricaño-Ponce *et al.* (2020), Stuart *et al.* (2022), and Wang *et al.* (2021).

** Published GWAS meta-analysis included UKBB; hence, summary statistics from Boer CG *et al.* (2021) were used in the analyses.
