# Supplementary material for: The phenotypic and genetic association between endometriosis and immunological diseases
Source: Hum Reprod. 2025 Apr 22;40(6):1195–209. doi: 10.1093/humrep/deaf062 (PMC12127507; doi:10.1093/humrep/deaf062)
Supplement: deaf062_Supplementary_Table_S6 [file deaf062_supplementary_table_s6.pdf]

OXFORD
human reproduction
Human Reproduction, 2025, 1–1
https://doi.org/10.1093/humrep/deaf062
Supplementary Data

Supplementary Table S6. Mendelian randomization (MR) results for endometriosis versus osteoarthritis, rheumatoid arthritis, and multiple sclerosis.

Immunological diseases	IVs	Inverse variance weighted		Weighted median		MR-Egger		MR-Egger (P-values)	
		OR (95% CI)	P-value	OR (95% CI)	P-value	OR (95% CI)	P-value	Heterogeneity[a]	Pleiotropy[b]
**Osteoarthritis**									
Female-only UKBB	39	1.04 (0.97–1.11)	0.282	1.05 (0.97–1.13)	0.267	0.89 (0.72–1.07)	0.203	0.002	0.084
Female-only UKBB (outliers removed)[c]	35	1.03 (0.98–1.09)	0.287	1.05 (0.97–1.13)	0.25	0.95 (0.79–1.15)	0.595	0.572	0.387
Combined-sex meta-analysis	39	1.02 (0.96–1.07)	0.533	1.02 (0.97–1.08)	0.4	1.08 (0.92–1.26)	0.353	1.56×10^{-5}	0.229
Combined-sex meta-analysis (outliers removed)[c]	36	1.01 (0.97–1.06)	0.495	1.03 (0.98–1.09)	0.281	1.03 (0.90–1.18)	0.655	0.159	0.8
**Rheumatoid arthritis**									
Female-only UKBB	39	1.16 (1.02–1.33)	0.028	1.15 (0.94–1.40)	0.169	1.15 (0.78–1.70)	0.494	0.756	0.959
Sex-combined meta-analysis	31	1.06 (0.96–1.17)	0.22	1.09 (0.94–1.26)	0.261	1.08 (0.76–1.53)	0.682	0.611	0.435
**Multiple sclerosis**									
Female-only UKBB	39	1.12 (0.87–1.43)	0.376	1.23 (0.87–1.73)	0.243	1.75 (0.84–3.64)	0.143	0.815	0.211
Combined-sex meta-analysis	39	1.06 (0.94–1.21)	0.343	1.03 (0.89–1.20)	0.692	1.24 (0.84–1.84)	0.28	0.012	0.145
Combined-sex meta-analysis (outliers removed)[c]	38	1.04 (0.93–1.18)	0.484	1.02 (0.88–1.2)	0.759	1.20 (0.84–1.73)	0.325	0.063	0.202

[a] Test for heterogeneity.
[b] Test for pleiotropy.
[c] Outlier IVs were identified by MR PRESSO software (see Materials and methods and Supplementary Table S7).
