# Supplementary material for: The phenotypic and genetic association between endometriosis and immunological diseases
Source: Hum Reprod. 2025 Apr 22;40(6):1195–209. doi: 10.1093/humrep/deaf062 (PMC12127507; doi:10.1093/humrep/deaf062)
Supplement: deaf062_Supplementary_Table_S7 [file deaf062_supplementary_table_s7.pdf]

OXFORD

human reproduction

Human Reproduction, 2025, 1–1
https://doi.org/10.1093/humrep/deaf062
Supplementary Data

Supplementary Table S7. MR PRESSO as sensitivity analysis for Mendelian randomization (MR) analysis investigating whether endometriosis (exposure) is causally associated with osteoarthritis (OA), rheumatoid arthritis (RA), or multiple sclerosis (MS) (outcome).

Immunological diseases	Model*	Causal estimate	SD	*t*-statistics	*P*-value	Global test (RSS_observed)	Global test (*P*-value)
RA	Raw	0.062	0.048	1.296	0.205	28.57	28.57
	Outlier corrected	NA	NA	NA	NA	NA	NA
OA	Raw	0.017	0.027	0.623	0.537	93.23	<0.001
	Outlier corrected	0.014	0.021	0.682	0.5	44.535	44.535
MS	Raw	−0.062	0.066	−0.948	0.349	66.916	0.006
	Outlier corrected	−0.043	0.061	−0.699	0.489	56.016	0.033

* Results by MR PRESSO as sensitivity checks on outliers are presented for MR analysis models before removing outliers (raw model) and after removing outliers (outlier-corrected models).
