# Supplementary material for: The phenotypic and genetic association between endometriosis and immunological diseases
Source: Hum Reprod. 2025 Apr 22;40(6):1195–209. doi: 10.1093/humrep/deaf062 (PMC12127507; doi:10.1093/humrep/deaf062)
Supplement: deaf062_Supplementary_Table_S8 [file deaf062_supplementary_table_s8.pdf]

OXFORD
human reproduction
Human Reproduction, 2025, 1–1
https://doi.org/10.1093/humrep/deaf062
Supplementary Data

Supplementary Table S8. Genome-wide significant ($P < 5 \times 10^{-8}$) lead single nucleotide polymorphisms (SNPs) associated with endometriosis in MTAG.

SNP	CHR	BP	EA	OA	Single trait association Z	EAF	MTAG beta	MTAG SE	MTAG Z	MTAG P-value	Novel
rs56319427	1	22396998	T	C	−13.419	0.832	−0.050	0.004	−13.431	3.94E-41	No
rs12030576	1	115817221	G	T	7.353	0.654	0.022	0.003	7.654	1.94E-14	No
rs2421985	1	172099136	T	C	−7.056	0.515	−0.021	0.003	−7.549	4.37E-14	No
rs11674184	2	11721535	T	G	10.093	0.610	0.030	0.003	10.403	2.39E-25	No
rs12467319	2	27348687	T	C	4.611	0.588	0.016	0.003	5.744	9.26E-09	Yes
rs11126143	2	67865408	C	T	5.533	0.325	0.018	0.003	6.188	6.08E-10	No
rs56397913	2	98599499	C	T	−4.816	0.922	−0.029	0.005	−5.520	3.38E-08	Yes
rs72928925	2	203554324	A	C	5.085	0.821	0.022	0.004	6.116	9.61E-10	No
rs6774202	3	49687779	T	G	5.511	0.174	0.025	0.004	6.811	9.70E-12	No
rs1903068	4	56008477	A	G	10.211	0.677	0.032	0.003	10.733	7.09E-27	No
rs72617705	4	95620225	G	T	−5.396	0.856	−0.024	0.004	−6.086	1.15E-09	No
rs4703918	5	82040476	A	G	−5.303	0.661	−0.017	0.003	−5.947	2.73E-09	Yes
rs2964485	5	157904839	G	T	−5.233	0.225	−0.019	0.003	−5.713	1.11E-08	No
rs6456259	6	19761718	A	G	−8.385	0.840	−0.033	0.004	−8.729	2.58E-18	No
rs4540228	6	74613060	C	T	6.487	0.666	0.020	0.003	6.835	8.18E-12	No
rs1777224	6	126019527	T	C	−5.986	0.559	−0.017	0.003	−6.093	1.11E-09	No
rs17215781	6	152570274	A	G	10.021	0.925	0.056	0.005	10.707	9.42E-27	No
rs11756073	6	170363580	G	A	−5.745	0.831	−0.022	0.004	−5.893	3.80E-09	No
rs1859164	7	27218419	T	C	−5.423	0.534	−0.017	0.003	−6.026	1.68E-09	No
rs940721	7	46796166	A	C	5.515	0.837	0.021	0.004	5.604	2.09E-08	No
rs34751086	7	94592691	C	T	5.229	0.876	0.023	0.004	5.529	3.23E-08	Yes
rs12542037	8	10758496	G	A	−4.972	0.555	−0.018	0.003	−6.520	7.02E-11	Yes
rs17053711	8	25311269	G	A	5.366	0.735	0.018	0.003	5.894	3.76E-09	No
rs10090060	8	75257608	A	G	7.589	0.570	0.021	0.003	7.525	5.28E-14	No
rs13275821	8	100084782	C	T	5.933	0.205	0.020	0.003	5.780	7.49E-09	No
rs2009382	8	116633906	C	T	4.973	0.387	0.017	0.003	5.819	5.92E-09	Yes
rs1537377	9	22169700	T	C	−8.205	0.607	−0.025	0.003	−8.837	9.83E-19	No
rs55724065	9	119471669	G	A	5.302	0.778	0.019	0.003	5.553	2.80E-08	No
rs507666	9	136149399	G	A	−5.427	0.807	−0.020	0.004	−5.734	9.80E-09	No
rs11012732	10	21830104	A	G	−6.920	0.662	−0.024	0.003	−8.143	3.84E-16	No
rs6586134	10	90202725	G	A	6.181	0.436	0.018	0.003	6.407	1.49E-10	No
rs3858429	11	30343757	C	T	11.320	0.843	0.045	0.004	11.681	1.59E-31	No
rs7924571	11	32350027	C	A	5.907	0.770	0.021	0.003	6.412	1.44E-10	No
rs28410854	12	15411810	A	G	−6.093	0.673	−0.020	0.003	−6.638	3.18E-11	No
rs12320196	12	95645385	A	G	−6.887	0.527	−0.019	0.003	−6.895	5.37E-12	No
rs7138106	12	102617138	G	A	5.882	0.823	0.021	0.004	5.748	9.02E-09	No
rs1028862	13	51055134	G	A	−5.471	0.862	−0.025	0.004	−6.115	9.63E-10	No
rs57281976	14	93112974	G	A	5.744	0.765	0.019	0.003	5.755	8.65E-09	No
rs12441483	15	40353416	T	C	−7.347	0.532	−0.021	0.003	−7.649	2.02E-14	No
rs67446770	17	46235745	A	C	−6.027	0.410	−0.018	0.003	−6.138	8.37E-10	No
rs7214750	17	63903119	C	T	6	0.930	0.033	0.005	6.082	1.19E-09	No
rs2164983	19	8789381	C	A	−5.667	0.842	−0.021	0.004	−5.617	1.95E-08	No

SNP: single nucleotide polymorphism, CHR: chromosome, BP: base-pair position, EA: effective allele, OA: other allele, EAF: effective allele frequency, SE: standard error of beta coefficient.
