# Supplementary material for: The phenotypic and genetic association between endometriosis and immunological diseases
Source: Hum Reprod. 2025 Apr 22;40(6):1195–209. doi: 10.1093/humrep/deaf062 (PMC12127507; doi:10.1093/humrep/deaf062)
Supplement: deaf062_Supplementary_Table_S10 [file deaf062_supplementary_table_s10.pdf]

OXFORD
human reproduction
Human Reproduction, 2025, 1–1
https://doi.org/10.1093/humrep/deaf062
Supplementary Data

Supplementary Table S10. Genome-wide significant ($P < 5 \times 10^{-8}$) lead single nucleotide polymorphisms (SNPs) associated with osteoarthritis in MTAG.

SNP	CHR	BP	EA	OA	Single trait association Z	EAF	MTAG beta	META SE	MTAG Z	MTAG P-value	Novel
rs785113	1	40154983	T	C	−5.354	0.241	−0.014	0.002	−5.699	1.20E-08	1
rs4098282	1	103470547	G	A	−6.72	0.1602	−0.018	0.003	−6.304	2.90E-10	2
rs4650948	1	174089158	T	C	5.565	0.7241	0.014	0.002	6.021	1.73E-09	3
rs58774073	2	33431618	C	T	7.273	0.5103	0.014	0.002	6.764	1.34E-11	4
rs858936	2	50940168	T	C	−5.44706	0.8792	−0.018	0.003	−5.607	2.06E-08	5
rs2443798	2	204394236	C	T	−6.018	0.4084	−0.014	0.002	−6.494	8.39E-11	6
rs10049087	3	50123417	A	G	5.909	0.5253	0.013	0.002	6.541	6.12E-11	7
rs12511923	4	1745152	C	T	6	0.811	0.015	0.003	5.641	1.70E-08	8
rs13107325	4	103188709	C	T	−8.829	0.9249	−0.034	0.004	−8.726	2.64E-18	9
rs72688751	4	130256802	C	T	−5.179	0.8522	−0.016	0.003	−5.668	1.44E-08	10
rs7731752	5	170802522	T	C	4.818	0.459	0.011	0.002	5.494	3.94E-08	11
rs6966540	7	95727967	T	C	−6.333	0.6265	−0.013	0.002	−6.049	1.46E-09	12
rs330088	8	9149746	T	C	−6.309	0.4532	−0.014	0.002	−6.941	3.90E-12	13
rs4240673	8	10787612	T	C	6.143	0.4516	0.015	0.002	7.019	2.24E-12	14
rs35851103	8	11869560	T	G	−6.109	0.5054	−0.014	0.002	−6.672	2.53E-11	15
rs3892354	9	4282942	T	G	−5.482	0.4132	−0.012	0.002	−5.587	2.31E-08	19
rs4979341	9	116905543	C	T	−7.629	0.7299	−0.018	0.002	−7.587	3.27E-14	20
rs12357321	10	21790476	G	A	−4.817	0.6879	−0.013	0.002	−5.917	3.28E-09	21
rs4757145	11	13331324	G	A	5.211	0.6185	0.012	0.002	5.646	1.64E-08	22
rs1149611	11	76495879	T	C	5.911	0.5715	0.012	0.002	5.69	1.27E-08	23
rs2171126	12	94167220	C	T	−6.164	0.4928	−0.012	0.002	−5.938	2.88E-09	24
rs4930719	12	123908948	G	A	5.493	0.8146	0.015	0.003	5.638	1.72E-08	25
rs35206230	15	75097780	C	T	−7.034	0.329	−0.015	0.002	−6.811	9.72E-12	26
rs258342	16	89694620	A	G	−5.738	0.2431	−0.014	0.002	−5.648	1.62E-08	27
rs10502437	18	20970706	G	A	5.526	0.6047	0.013	0.002	5.985	2.16E-09	28
rs1560707	19	10750738	T	G	7.421	0.3706	0.015	0.002	7.172	7.38E-13	29
rs224333	20	34023962	G	A	7.456	0.6393	0.016	0.002	7.51	5.90E-14	30

SNP: single nucleotide polymorphism, CHR: chromosome, BP: base-pair position, EA: effective allele, OA: other allele, EAF: effective allele frequency, SE: standard error of beta coefficient.
