# Supplementary material for: The phenotypic and genetic association between endometriosis and immunological diseases
Source: Hum Reprod. 2025 Apr 22;40(6):1195–209. doi: 10.1093/humrep/deaf062 (PMC12127507; doi:10.1093/humrep/deaf062)
Supplement: deaf062_Supplementary_Table_S11 [file deaf062_supplementary_table_s11.pdf]

OXFORD
human reproduction

Human Reproduction, 2025, 1–1
https://doi.org/10.1093/humrep/deaf062
Supplementary Data

Supplementary Table S11. Genome-wide significant ($P < 5 \times 10^{-8}$) lead single nucleotide polymorphisms (SNPs) associated with rheumatoid arthritis in MTAG.

SNP	CHR	BP	EA	OA	Single trait association Z	EAF	MTAG beta	META SE	MTAG Z	MTAG P-value	Novel
rs867436	1	2523723	C	T	5.352	0.356	0.031	0.005	6.25	4.10E-10	1
rs1635578	1	17666095	T	C	5.775	0.321	0.029	0.005	5.681	1.34E-08	2
rs6679677	1	114303808	C	A	−21.626	0.102	−0.16	0.008	−20.4	1.61E-92	3
rs906868	2	30448344	G	T	−5.824	0.635	−0.028	0.005	−5.713	1.11E-08	4
rs13026755	2	61160619	C	T	−5.74	0.345	−0.031	0.005	−6.192	5.94E-10	5
rs3024886	2	191943742	C	T	−7.253	0.225	−0.038	0.006	−6.717	1.85E-11	6
rs3087243	2	204738919	G	A	6.806	0.452	0.028	0.005	5.969	2.39E-09	7
rs7617779	3	17045586	A	G	−5.712	0.73	−0.03	0.005	−5.65	1.60E-08	8
rs34269949	3	27769551	A	C	−4.752	0.548	−0.029	0.005	−6.17	6.85E-10	9
rs16878091	4	26088128	G	A	−8.152	0.304	−0.038	0.005	−7.41	1.26E-13	10
rs7731626	5	55444683	G	A	10.664	0.376	0.052	0.005	10.554	4.89E-26	11
rs6920220	6	138006504	G	A	−8.226	0.222	−0.04	0.006	−7.016	2.29E-12	12
rs968334	6	167526096	T	C	7.048	0.443	0.031	0.005	6.387	1.69E-10	13
rs10261758	7	50353192	G	A	5.676	0.712	0.029	0.005	5.539	3.05E-08	14
rs3757387	7	128576086	T	C	−7.397	0.548	−0.037	0.005	−7.82	5.30E-15	15
rs2736340	8	11343973	C	T	−5.217	0.255	−0.032	0.005	−5.944	2.78E-09	16
rs706778	10	6098949	C	T	−6.672	0.401	−0.036	0.005	−7.509	5.94E-14	17
rs11593907	10	63786554	T	C	−6.973	0.792	−0.035	0.006	−6.004	1.92E-09	18
rs7117261	11	118741157	T	C	−6.038	0.193	−0.04	0.006	−6.675	2.47E-11	19
rs9603618	13	40371377	C	T	5.718	0.281	0.031	0.005	5.882	4.05E-09	20
rs72727387	15	38843476	G	A	−6.863	0.259	−0.039	0.005	−7.135	9.69E-13	21
rs17374222	15	69995344	C	A	−7.448	0.493	−0.036	0.005	−7.532	4.98E-14	22
rs7201780	16	30147265	C	T	4.956	0.441	0.029	0.005	6.155	7.50E-10	23
rs9933582	16	86016026	T	G	−5.422	0.769	−0.034	0.006	−5.956	2.58E-09	24
rs12941333	17	38040534	C	T	−5.562	0.477	−0.026	0.005	−5.508	3.63E-08	25
rs2278442	19	10444826	G	A	−6.328	0.645	−0.033	0.005	−6.65	2.94E-11	26
rs2409493	21	34808289	A	G	5.724	0.537	0.027	0.005	5.698	1.21E-08	27
rs2069235	22	39747780	G	A	−5.622	0.3	−0.03	0.005	−5.774	7.75E-09	28

SNP: single nucleotide polymorphism, CHR: chromosome, BP: base-pair position, EA: effective allele, OA: other allele, EAF: effective allele frequency, SE: standard error of beta coefficient.
