# Supplementary material for: The phenotypic and genetic association between endometriosis and immunological diseases
Source: Hum Reprod. 2025 Apr 22;40(6):1195–209. doi: 10.1093/humrep/deaf062 (PMC12127507; doi:10.1093/humrep/deaf062)
Supplement: deaf062_Supplementary_Table_S12 [file deaf062_supplementary_table_s12.pdf]

OXFORD
human reproduction
Human Reproduction, 2025, 1–2
https://doi.org/10.1093/humrep/deaf062
Supplementary Data

Supplementary Table S12. Genome-wide significant ($P < 5 \times 10^{-8}$) lead single nucleotide polymorphisms (SNPs) associated with multiple sclerosis in MTAG.

SNP	CHR	BP	EA	OA	Single trait association Z	EAF	MTAG beta	META SE	MTAG Z	MTAG P-value	Novel
rs6701238	1	2520500	G	A	8.22	0.657	0.063	0.007	8.681	3.91E-18	1
rs10873698	1	85756250	A	G	−5.86	0.626	−0.04	0.007	−5.658	1.53E-08	2
rs11809700	1	93152635	C	T	−7.871	0.733	−0.062	0.008	−7.958	1.74E-15	3
rs61780263	1	101291168	A	C	7.589	0.912	0.0932	0.012	7.656	1.92E-14	4
rs10754324	1	117093035	T	C	8.076	0.869	0.082	0.01	8.028	9.90E-16	5
rs532303	1	120265444	A	G	−5.985	0.316	−0.043	0.007	−5.716	1.09E-08	6
rs1323292	1	192541021	G	A	−5.673	0.18	−0.051	0.009	−5.725	1.03E-08	7
rs59655222	1	200875897	T	C	6.613	0.722	0.047	0.008	6.067	1.30E-09	8
rs28411734	2	43355519	A	G	6.513	0.722	0.051	0.008	6.575	4.87E-11	9
rs1177228	2	61242410	A	G	−5.757	0.266	−0.045	0.008	−5.794	6.86E-09	10
rs12622670	2	68646536	C	T	−6.461	0.459	−0.042	0.007	−6.039	1.56E-09	11
rs57116599	2	112770799	G	A	5.956	0.774	0.048	0.008	5.814	6.09E-09	12
rs7584894	2	231091656	G	A	−6.819	0.825	−0.06	0.009	−6.545	5.94E-11	13
rs438613	3	28072086	T	C	−8.312	0.523	−0.059	0.007	−8.491	2.06E-17	14
rs34844599	3	119252208	T	C	6.171	0.825	0.056	0.009	6.204	5.52E-10	15
rs2681424	3	121769522	T	C	7.308	0.508	0.05	0.007	7.189	6.50E-13	16
rs1014486	3	159691112	T	C	−6.42	0.563	−0.045	0.007	−6.391	1.65E-10	17
rs6854803	4	109095552	G	A	5.399	0.422	0.038	0.007	5.481	4.22E-08	18
rs6881270	5	35879095	C	T	6.075	0.73	0.047	0.008	6.058	1.38E-09	19
rs11749040	5	40396425	G	A	−8.427	0.867	−0.085	0.01	−8.31	9.53E-17	20
rs7731626	5	55444683	G	A	4.682	0.629	0.042	0.007	5.862	4.58E-09	21
rs2546890	5	158759900	A	G	7.125	0.522	0.049	0.007	7.166	7.71E-13	22
rs72928038	6	90976768	G	A	−6.483	0.824	−0.061	0.009	−6.756	1.41E-11	23
rs802730	6	128280104	T	C	6.287	0.699	0.044	0.008	5.881	4.08E-09	24
rs13197384	6	135818897	C	A	−7.323	0.651	−0.054	0.007	−7.408	1.28E-13	25
rs62420820	6	137438057	G	A	−7.319	0.76	−0.058	0.008	−7.098	1.27E-12	26
rs1738074	6	159465977	T	C	−6.808	0.422	−0.049	0.007	−7.066	1.59E-12	27
rs55970742	7	2441337	C	T	5.608	0.322	0.041	0.007	5.561	2.69E-08	28
rs4739134	8	79556148	T	C	5.688	0.267	0.046	0.008	5.845	5.06E-09	29
rs6990534	8	128814091	A	G	−5.902	0.297	−0.045	0.008	−5.994	2.05E-09	30
rs7078535	10	6120424	C	T	−9.756	0.439	−0.068	0.007	−9.77	1.52E-22	31
rs1250551	10	81059335	G	T	−6.664	0.644	−0.049	0.007	−6.725	1.76E-11	32
rs1112718	10	94479107	A	G	6.329	0.599	0.045	0.007	6.406	1.49E-10	33
rs4939491	11	60793722	G	A	−7.846	0.601	−0.052	0.007	−7.3	2.88E-13	34
rs12365699	11	118743286	G	A	6.291	0.831	0.063	0.009	6.834	8.28E-12	35
rs1800693	12	6440009	T	C	−7.439	0.591	−0.051	0.007	−7.266	3.70E-13	36
rs7977720	12	9866349	C	T	−6.256	0.535	−0.041	0.007	−5.923	3.16E-09	37
rs701006	12	58106836	A	G	−6.763	0.405	−0.049	0.007	−7.037	1.96E-12	38
rs7975763	12	123604053	C	T	−5.773	0.787	−0.046	0.008	−5.482	4.21E-08	39
rs9591325	13	50811220	T	C	6.248	0.926	0.085	0.013	6.396	1.59E-10	40
rs12435329	14	69250891	T	C	6.331	0.535	0.039	0.007	5.674	1.40E-08	41
rs34695601	14	76014298	T	C	5.532	0.76	0.046	0.008	5.708	1.15E-08	42
rs17124032	14	88546009	G	A	6.856	0.92	0.089	0.013	6.971	3.15E-12	43
rs12147246	14	103265844	A	G	5.872	0.349	0.044	0.007	6.092	1.12E-09	44
rs6496664	15	90888561	A	G	−5.542	0.689	−0.042	0.007	−5.657	1.54E-08	45
rs415759	16	1066917	T	C	−5.46	0.812	−0.049	0.009	−5.607	2.05E-08	46
rs7200786	16	11177801	A	G	10.045	0.456	0.068	0.007	9.839	7.62E-23	47
rs3809627	16	30103160	C	A	5.528	0.586	0.04	0.007	5.671	1.42E-08	48
rs12925972	16	79111297	T	C	−5.537	0.455	−0.038	0.007	−5.466	4.60E-08	49
rs9933582	16	86016026	T	G	−5.117	0.777	−0.047	0.008	−5.619	1.92E-08	50
rs1026916	17	40529835	A	G	7.438	0.353	0.055	0.007	7.667	1.76E-14	51
rs7216796	17	43392208	G	A	−5.376	0.461	−0.038	0.007	−5.494	3.92E-08	52
rs11079784	17	45702280	T	C	−6.592	0.499	−0.044	0.007	−6.363	1.97E-10	53
rs2150879	17	57859210	G	A	6.285	0.456	0.044	0.007	6.307	2.84E-10	54
rs11873030	18	56389432	C	T	5.615	0.764	0.048	0.008	5.865	4.49E-09	55
rs1077667	19	6668972	C	T	7.155	0.792	0.058	0.009	6.858	7.01E-12	56
rs28834106	19	10592144	T	C	6.612	0.716	0.053	0.008	6.92	4.52E-12	57
rs874628	19	18304700	A	G	7.219	0.715	0.057	0.008	7.379	1.60E-13	58
rs8102445	19	47637608	G	T	5.878	0.442	0.039	0.007	5.694	1.24E-08	59

(continued)

Supplementary Table S12. Continued

SNP	CHR	BP	EA	OA	Single trait association Z	EAF	MTAG beta	META SE	MTAG Z	MTAG P-value	Novel
rs1465697	19	49837246	C	T	−6.625	0.738	−0.052	0.008	−6.552	5.66E-11	60
rs13037326	20	44692598	C	T	−7.248	0.734	−0.052	0.008	−6.588	4.45E-11	61
rs3935549	20	47253487	T	C	−5.532	0.517	−0.038	0.007	−5.521	3.36E-08	62
rs2259735	20	52788314	T	C	6.027	0.577	0.04	0.007	5.754	8.73E-09	63
rs9610458	22	22205353	C	T	−6.918	0.464	−0.047	0.007	−6.819	9.19E-12	64

SNP: single nucleotide polymorphism, CHR: chromosome, BP: base-pair position, EA: effective allele, OA: other allele, EAF: effective allele frequency, SE: standard error of beta coefficient.

© The Author(s) 2025. Published by Oxford University Press on behalf of European Society of Human Reproduction and Embryology.
This is an Open Access article distributed under the terms of the Creative Commons Attribution License (https://creativecommons.org/licenses/by/4.0/), which permits unrestricted reuse, distribution, and reproduction in any medium, provided the original work is properly cited.
Human Reproduction, 2025, 12, 1–47
https://doi.org/10.1093/humrep/deaf062
Original Article
